# Polysome profiling reveals translational control of gene expression in the human malaria parasite *Plasmodium falciparum*

**DOI:** 10.1186/gb-2013-14-11-r128

**Published:** 2013-11-22

**Authors:** Evelien M Bunnik, Duk-Won Doug Chung, Michael Hamilton, Nadia Ponts, Anita Saraf, Jacques Prudhomme, Laurence Florens, Karine G Le Roch

**Affiliations:** 1 Department of Cell Biology and Neuroscience, University of California Riverside, 900 University Ave, Riverside, CA 92521, USA; 2 Current address: INRA Centre de Bordeaux Aquitaine, 33883 Villenave d’Ornon, Cedex, France; 3 Stowers Institute for Medical Research, Kansas City, MO 64110, USA; 4 Institute for Integrative Genome Biology, Center for Disease Vector Research, Department of Cell Biology and Neuroscience, University of California Riverside, 900 University Avenue, Riverside, CA 92521, USA

## Abstract

**Background:**

In eukaryotic organisms, gene expression is regulated at multiple levels during the processes of transcription and translation. The absence of a tight regulatory network for transcription in the human malaria parasite suggests that gene expression may largely be controlled at post-transcriptional and translational levels.

**Results:**

In this study, we compare steady-state mRNA and polysome-associated mRNA levels of *Plasmodium falciparum* at different time points during its asexual cell cycle. For more than 30% of its genes, we observe a delay in peak transcript abundance in the polysomal fraction as compared to the steady-state mRNA fraction, suggestive of strong translational control. Our data show that key regulatory mechanisms could include inhibitory activity of upstream open reading frames and translational repression of the major virulence gene family by intronic transcripts. In addition, we observe polysomal mRNA-specific alternative splicing events and widespread transcription of non-coding transcripts.

**Conclusions:**

These different layers of translational regulation are likely to contribute to a complex network that controls gene expression in this eukaryotic pathogen. Disrupting the mechanisms involved in such translational control could provide novel anti-malarial strategies.

## Background

Malaria is still one of the most deadly infectious diseases worldwide, claiming an estimated 660,000 lives per year [1]. The vast majority of deaths occur among children under the age of 5 years living in sub-Saharan Africa [1]. Over the past decade, malaria control measures have reduced the global incidence and mortality rates by 17% and 26%, respectively [1]. However, the absence of a preventive vaccine and the spread of drug-resistant parasite strains warrant continued investigations into the intricate biology of the malaria parasite, in search of novel anti-malarial drug targets.

The malaria parasite species *Plasmodium falciparum* is responsible for 90% of all malaria deaths [1]. The complex life cycle of *P. falciparum* involves multiple stages in both the human and the mosquito host. The symptomatic phase of *P. falciparum* infection is the erythrocytic stage, where the parasite replicates in red blood cells and progresses through the ring, trophozoite and schizont stages to produce 16 to 32 daughter cells. The release of these daughter cells, or merozoites, into the blood stream after the completion of each 48-hour cycle of cell division causes the typical pattern of recurring fevers. Environmental stress, such as low nutrient levels, induces the formation of gametocytes, the sexual forms of *P. falciparum*, which can be transferred to a mosquito host when it takes a blood meal.

The multiplication process during the erythrocytic cell cycle of *P. falciparum* infection is tightly regulated and involves the expression of the majority of its genes [2-4]. However, the regulation of gene expression in *P. falciparum* is still incompletely understood. Relatively few transcription factors have been identified [5,6], while changes in chromatin structure seem to play a unique role in transcriptional control [7,8]. Moreover, for a large proportion of genes expressed in the erythrocytic cycle, transcriptional activity does not correlate well with protein abundance [9,10], similar to mammalian cells where the initiation of translation, and not transcript abundance, is the main determinant of protein levels [11]. In *Plasmodium berghei* gametocytes, delayed translation of two transcripts was shown to occur by temporary storage of these transcripts in P-bodies, followed by transfer to ribosomes after ingestion of gametocytes by a mosquito [12]. RNA-binding proteins are likely to be involved in translational repression at this stage [13]. In addition, latency of *P. berghei* sporozoites is controlled by phosphorylation of eukaryotic initiation factor-2α, resulting in inhibition of translation [14]. However, the mechanisms and the extent of post-transcriptional and translational control have not yet been described for the asexual stage of *P. falciparum*.

In other eukaryotic organisms, a multitude of mechanisms act in concert to regulate gene expression at a post-transcriptional level, including mRNA splicing, decay, binding of inhibitory proteins and the actions of regulatory mRNA elements. One of the major regulatory mechanisms of mRNA abundance in higher eukaryotes is RNA interference, but homologues of the RNA interference machinery have not been identified in the *P. falciparum* genome [15].

In this study, we performed next-generation sequencing of both steady-state mRNA and polysome-associated mRNA, presumed to be actively translated. Our genome-wide approach allowed us to elucidate the extent of translational control during the erythrocytic cell cycle of *P. falciparum* and to identify key mechanisms likely contributing to the complex regulatory network of gene expression and parasite virulence. Collectively, our results increase our understanding of parasite development throughout the infectious cell cycle, which may contribute to novel antimalarial strategies.

## Results

### Generation of steady-state mRNA and polysomal mRNA datasets across the *P. falciparum* asexual cycle

**Figure 1:**
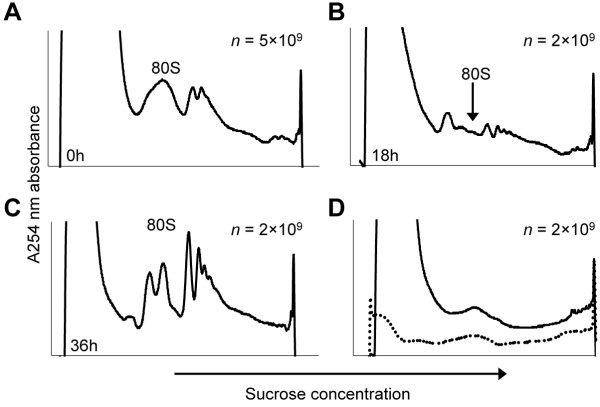
**Polysome profiles from different time points during the erythrocytic cycle of *P. falciparum*. (A-C)** Polysome sucrose (15 to 60%) absorbance (A_254_ nm) profiles obtained from erythrocytes infected with ring-stage **(A)**, trophozoite-stage **(B)**, or schizont-stage **(C)** parasites. **(D)** Profiles obtained from an uninfected erythrocyte culture (black line) and a blank gradient (dotted line). The number of parasites used to obtain each profile is indicated in the top right corner of the graph. For the uninfected culture in **(D)**, the amount of culture used was equal to the amount of infected culture containing the indicated number of parasites. The monosome peak is marked as 80S.

To investigate differences between transcription and translation during the erythrocytic cycle of *P. falciparum* strain 3D7, we isolated both steady-state mRNA and polysome-associated mRNA at different stages throughout the parasite’s cell cycle. Parasites were harvested directly after the invasion of the red blood cell at the early ring stage (0 h), as well as at the trophozoite (18 h) and schizont (36 h) stages. For steady-state mRNA, we first isolated total RNA from the parasites, followed by mRNA purification using poly-A selection. Based on the amounts of mRNA isolated per flask of parasites, high abundance of transcripts was observed during the trophozoite and schizont stages of the erythrocytic cycle (Table 1). For polysomal mRNA, we isolated polysomes by sucrose density gradient centrifugation [16], also followed by mRNA purification using poly-A selection. Translational activity peaked at the schizont stage (Table 1; Figure 1). Polysomes were absent in a profile from cultured uninfected erythrocytes, indicating that contamination levels of human ribosomes in polysome isolations from *P. falciparum* cultures were very low (Figure 1D). In addition, to validate the selectivity of our polysome isolation procedure, we analyzed polysome fractions by highly sensitive, semi-quantitative mass-spectrometry (multidimensional protein identification technology; MudPIT [17]). Our analysis yielded 95.6% ribosomal proteins, 2.0% RNA-binding and ribosome-associated proteins (such as translation initiation factors, elongation factors, and other RNA-binding proteins), 0.5% proteins with unknown functions and 2.0% contaminants (Table S1 in Additional file 1), indicative of a high purity of our polysome fractions. Both steady-state and polysomal mRNA fractions were treated with DNase to remove genomic DNA contamination. Even though steady-state mRNA contains all of the polysome-associated mRNA, the polysomal fraction is presumed to be highly enriched for actively translated mRNA and is thus considered a different mRNA population. Using next-generation sequencing (Illumine HiSeq 2000), we obtained an average of 7.1 and 3.0 million high quality reads per stage for the steady-state mRNA and polysomal mRNA datasets, respectively, corresponding to an average of 21.5X and 6.6X exome-wide coverage (Table 1). We observed a high correlation for gene expression values of biological replicates (Pearson R = 0.90; Figure S1A in Additional file 2), confirming the reproducibility of our methodology. Genome-wide gene expression levels also correlated well between our steady-state mRNA-Seq dataset and previously published microarray (R > 0.54) and RNA-Seq datasets (R > 0.70) [3,4,18] (Figure S1B,C in Additional file 2). Considering the fact that microarray and RNA-Seq data only agree well for genes with medium expression levels [19], the correlation coefficients that we obtained for our datasets further validate our sequencing results.

**Table 1:** Number of working reads and normalization factor for each library

**mRNA fraction**	**Time point (hours post-invasion)**	**mRNA quantity per 10**^**9 **^**parasites (μg)**	**Number of working reads**	**Exome-wide coverage**	**Scaling factor**
Steady-state	0 h	0.56	7,663,318	22.0X	5.3
	18 h	1.89	8,786,721	27.2X	1.8
	36 h	1.90	4,942,888	15.4X	1.0
Polysomal	0 h	0.07	438,272	0.69X	1.0
	18 h	0.24	5,708,551	12.7X	4.0
	36 h	0.33	2,809,130	6.3X	1.4
	36 h - biological replicate	0.32	846,855	1.9X	NA

NA, not applicable.

To compare gene expression levels between the different stages of the asexual cycle, we normalized the total sequence read counts of each library to directly reflect the mRNA levels per parasite at that stage. This was achieved by introducing a scaling factor for the sequence read counts per gene based on the total amount of mRNA isolated per flask (see Materials and methods; Table 1). After normalization, the distribution of read counts per gene for the trophozoite and schizont time points was relatively similar, while the average expression level of genes at the ring stage was much lower (Figure S2 in Additional file 2), corresponding to relative mRNA levels per parasite for each stage.

### Cluster analysis of steady-state and polysomal mRNA profiles across the asexual cell cycle

**Figure 2:**
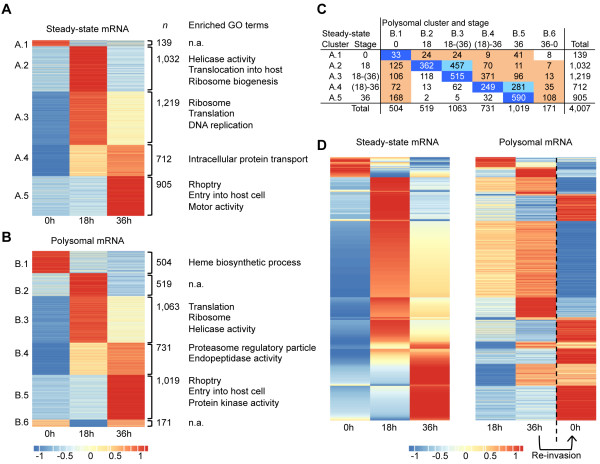
**Steady-state mRNA and polysome-association mRNA profiles of genes expressed in the asexual stage of *P. falciparum*. (A)** Cluster analysis of 4,007 genes based on steady-state mRNA accumulation. **(B)** Independent cluster analysis of the same set of genes from **(A)** based on polysome-associated mRNA accumulation. For each cluster, the number of genes (*n*) and a selection of enriched gene ontology (GO) terms are presented. **(C)** Number of genes that were found in each combination of steady-state mRNA and polysomal mRNA clusters. Combinations of identical transcription and translation profiles are indicated in dark blue (1,749 genes), while combinations with a partial translational delay are indicated in light blue (738 genes). For 1,280 genes, a delay between the time point of transcript accumulation and the moment of peak translation was observed (shown in light orange). **(D)** Steady-state mRNA and polysomal mRNA profiles of genes that showed a delay in polysome-association as compared to the time point of transcript accumulation (*n* = 1,280). Genes are ordered identically in both heatmaps to allow a comparison of steady-state mRNA and polysomal mRNA profiles. To visualize the delay of genes from the mature stages to re-invaded ring stage parasites, the 0 h time point of the next cell cycle is displayed for polysome-associated mRNA. N.a., not available.

We detected a total of 4,633 expressed genes (85.0% of all protein-coding genes) in our datasets, albeit at very low levels for some genes, which is comparable to previous expression analyses in *P. falciparum*[3,4]. Genome-wide, both steady-state mRNA levels and polysomal mRNA levels varied over five orders of magnitude, while expression levels for individual genes across the cell cycle ranged within three orders of magnitude. Median variation in expression levels between different time points of the cell cycle were 3.8-fold for steady-state mRNA and 3.6-fold for polysomal mRNA. Of the genes that were present in both the steady-state mRNA and the polysome-associated mRNA datasets, 4,007 genes (73.5%) were cell cycle-regulated (defined as more than two-fold difference in expression levels across the cell cycle). Both the steady-state and the polysomal transcript abundance data were independently clustered based on expression profiles, resulting in five steady-state mRNA and six polysomal mRNA clusters (Figure 2). Most transcripts accumulated or were actively translated exclusively in one stage (clusters 1, 2, and 5 of the two datasets). However, for both the steady-state mRNA and polysomal mRNA datasets, we also identified a substantial number of genes with expression profiles overlapping the trophozoite and schizont stages (clusters 3 and 4). For most of these genes, expression peaked in one of the two stages with lower expression levels in the second stage. In addition, we observed a cluster of 171 genes that were associated with polysomes in both the schizont and the ring stage (cluster B.6). Gene ontology (GO) analysis showed large and coordinated shifts in the expression of groups of genes with common biological function in major cellular processes as the parasite progressed through its cell cycle (Figure 2; Tables S2 and S3 in Additional file 2). In preparation for translating the majority of its genes at the mature stages, genes involved in the process of translation, such as ribosomal proteins, were abundant and highly translated at the 18 h time point (clusters A.2, A.3, B.2, and B.3). A peak in transcript abundance of genes related to DNA replication was observed in the cluster peaking at the trophozoite stage but overlapping the schizont stage (cluster A.3), corresponding to the period of massive DNA replication that takes place as the parasite divides into daughter cells. Finally, transcripts for genes involved in invasion of a new host cell, such as rhoptry proteins, were highest in both the steady-state and polysome fraction from the schizont stage (clusters A.5 and B.5; Figure 2), just before merozoites are released into the blood stream to invade new red blood cells. The polysomal mRNA cluster analysis additionally showed enrichment of genes involved in heme biosynthetic process at the ring stage (cluster B.1) and of genes associated with protein degradation at the schizont stage (cluster B.4) (Figure 2B).

### Comparison of steady-state mRNA and polysomal mRNA profiles across the cell cycle

A comparison of steady-state mRNA and polysomal mRNA expression clusters revealed that for 1,749 genes (43.6%), transcription and translation showed similar patterns of upregulation, with transcripts peaking at the same time points in both fractions. For another 738 genes (18.4%), we observed a partial delay in the timing of translation as compared to the moment of transcription. For example, 457 genes were highly abundant in steady-state mRNA at the trophozoite stage but absent at the schizont stage (cluster A.2), while their abundance in polysomal mRNA peaked at the trophozoite stage and continued to the schizont stage (cluster B.3). Considering the 18 h window between the trophozoite and schizont time points, these partial shifts between steady-state mRNA and polysomal mRNA profiles are likely to be biologically relevant. In addition, we identified 1,280 genes (31.9%) for which translation was markedly delayed compared to the time point of transcription (Figure 2C,D). Among genes for which translation was delayed until the schizont stage, we found enrichment of genes involved in energy production (ATP synthesis-coupled proton transport; *P* = 1.2E-06).

More importantly, a substantial number of genes (*n* = 471) was found to be transcribed in the trophozoite and/or schizont stages of the cell cycle, while they were most highly associated with polysomes in the ring stage, suggesting that these transcripts are temporarily stored in the parasite and are not translated until after invasion of a new host cell. Among others, this group contained many genes involved in erythrocyte remodeling, such as members of the FIKK kinase family (*n* = 2 out of 16), the Maurer’s cleft two transmembrane (MC-2TM) protein family (*n* = 7 out of 11, *P* = 0.0027), Ring-infected erythrocyte surface antigen (RESA)-like proteins (*n* = 1 out of 4) and genes whose products are exported to the surface of the infected red blood cell (*n* = 31 out of 118, *P* < 0.0001; Table 2). The first two protein families localize to the Maurer’s cleft, a parasite structure in the cytoplasm of the infected erythrocyte necessary for trafficking of exported proteins to the cell surface. In addition, we observed a delay in translation for genes involved in metabolism, such as beta-ketoacyl-acyl carrier protein reductase (FabG; PF3D7_0922900) and acyl-CoA synthetase (ACS6; PF3D7_0401900). For FabG, the ratio of transcripts in steady-state versus polysome fractions was 1:47 at the ring stage, 2.6:1 at the trophozoite stage, and 3.5:1 at the schizont stage, while ACS6 showed ratios of 1:2.7, 2.2:0 and 2.2:1 at ring, trophozoite and schizont stages, respectively. Finally, based on our cluster analysis, a translational delay at any stage during the cell cycle was observed for seven out of 18 ApiAP2 transcription factors, 11 out of 11 key regulatory proteins encoded by the apicoplast, an organelle specific to apicomplexan organisms, and 565 out of 1,697 conserved *Plasmodium* proteins with unknown function (Table 2).

**Table 2:** Number of translationally delayed genes for selected groups of parasite-specific proteins

			**Average normalized read count per 1 kb exon (delay)**			**Average normalized read count per 1 kb exon (no delay)**		
**Group**	**Delay**^**a **^**( *n *)**	**Delay to R stage**^**b **^**( *n *)**	**Steady-state mRNA**	**Polysomal mRNA**	**No delay ( *n *)**	**Steady-state mRNA**	**Polysomal mRNA**	***P*-value**^**c**^
FIKK kinase family	8	2	113	25	8	232	30	-
MC-2TM	7	7	39	18	4	209	72	0.0027
RESA-like protein	2	1	23	9	2	71	12	-
Plasmepsin	5	3	251	40	3	1,913	113	-
Exported proteins	47	31	559	66	71	1,234	258	<0.001
ApiAP2 transcription factor	7	1	354	67	11	353	28	-
Apicoplast-encoded proteins	11	11	110	97	0	NA	NA	<0.001
Apicoplast-targeted proteins	120	50	420	90	255	969	283	-
Conserved proteins, unknown function	565	237	340	89	1,132	514	166	-
Total^d^	1,280	471	515	114	2,727	832	216	NA

^a^Defined as at least 18 h delay in peak abundance levels in polysomal mRNA as compared to steady-state mRNA.

^b^Subset of delayed genes, showing peak steady-state mRNA abundance in the trophozoite or schizont stage and peak polysomal mRNA abundance in the ring stage.

^c^Bonferroni-corrected two-tailed Fisher’s exact test for number of genes showing translational delay to the ring stage versus the number of non-delayed genes per group. *P*-values >0.01 are indicated with a dash.

^d^Total number of expressed and cell-cycle regulated genes included in our analysis.

NA., not applicable.

### Differences in mRNA landscape between steady-state mRNA and polysomal mRNA

**Figure 3:**
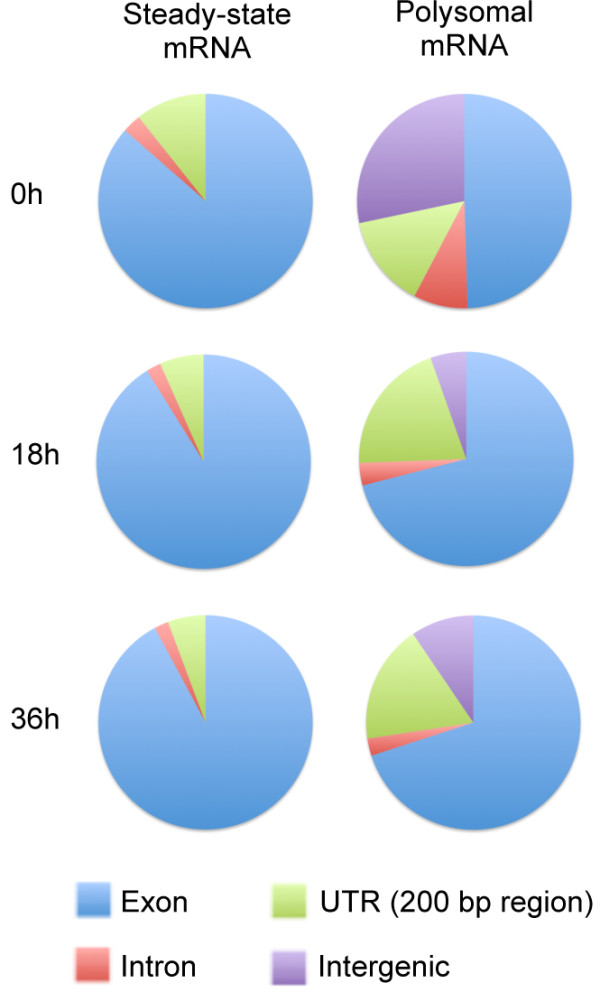
**Landscape of steady-state and polysomal mRNA.** The distribution of sequence reads over exons, introns, untranslated regions (UTRs; here defined as the 200 nucleotides directly upstream and downstream of the annotated start and stop sites of the coding sequence), and other intergenic regions is shown.

To identify mechanisms that may be involved in translational control, we compared genome coverage of sequence reads between our steady-state mRNA and polysome-associated mRNA datasets throughout the parasite cell cycle. The steady-state and polysomal mRNA landscapes were strikingly different (Figure 3). For steady-state mRNA, more than 85% of sequence reads mapped to coding sequence (CDS) regions, while between 29 and 50% of the polysomal mRNA reads mapped to introns, untranslated regions (UTRs) or other intergenic sequences. In particular, the low level of translation at the 0 h time point may allow us to detect non-annotated transcripts from intergenic regions present at very low levels, which could be overshadowed by highly translated genes at the later time points. To further investigate these differences, we studied the 5′ UTR, the 3′ UTR and the intronic regions in more detail, as addressed in the sections below.

### Control of translation by upstream open reading frames

**Figure 4:**
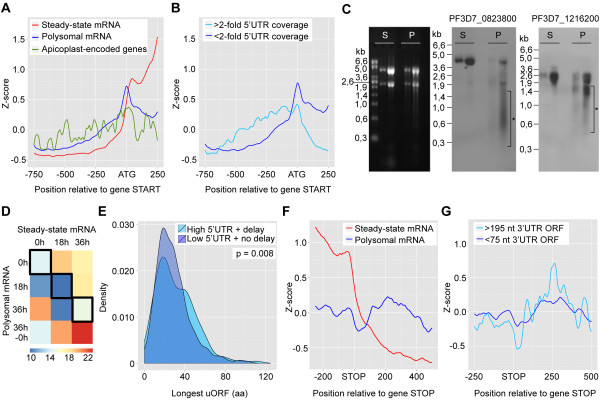
**Increased 5′ UTR and 3′ UTR coverage in the polysomal mRNA. (A)** Comparison of genome-wide 5′ UTR coverage at the 18 h time point between steady-state mRNA (red), polysomal mRNA (blue) and the subset of apicoplast-encoded genes in polysomal mRNA (green). **(B)** Increased 5′ UTR coverage in a subset of genes in polysomal mRNA. **(C)** Gel electrophoresis of steady-state RNA (S) and polysome-associated RNA (P) (left) from trophozoite-stage parasites, and northern blots of the same RNA fractions using a probe designed to detect sense transcripts containing the 5′ UTR of gene PF3D7_0823800 (middle), or gene PF3D7_1216200 (right). Smaller transcript fragments that are observed in the polysomal fraction but not in the steady-state fraction are marked with an asterisk. **(D)** Heatmap showing the percentage of genes with high 5′ UTR coverage out of the total number of genes for each combination of steady-state mRNA and polysomal mRNA profiles. Combinations where transcription and translation peak at the same time point are boxed. For clarity, expression profiles were grouped based on the time point of highest expression (for example, expression profiles 18 h and 18 h to (36 h) were grouped as 18 h). **(E)** Distribution of the longest upstream open reading frame (uORF) in genes that are expressed at the 18 h stage and that show high 5′ UTR coverage and translational delay, in comparison to genes that have low 5′ UTR coverage and no translational delay. The difference in distribution was tested for statistical significance using the Kolmogorov-Smirnov test. **(F)** Comparison of genome-wide 3′ UTR coverage at the 18 h time point between steady-state mRNA (red) and polysomal mRNA (blue). **(G)** Increased 3′ UTR coverage in polysomal mRNA for genes with a large ORF directly downstream of the stop codon (encoding a >65 amino acid (aa) peptide) as compared to genes with short (<25 amino acids) downstream ORFs, suggestive of stop codon readthrough.

In the polysomal mRNA dataset, a coverage plot of the start region averaged across all genes showed an increased coverage of the 5′ UTR region in combination with a lower coverage in the CDS (Figure 4A). In total, 409 genes showed more than two-fold read coverage in their 5′ UTR as compared to their CDS (Figure 4B). For three genes, this increased 5′ UTR coverage was validated by semi-quantitative RT-PCR, indicating that this phenomenon is unlikely to be the result of a bias introduced by the library preparation or sequencing reaction (Figure S3A in Additional file 2). In addition, reverse transcription using directional primers showed that mRNA covering the 5′ UTR was transcribed in the sense direction (Figure S3B in Additional file 2). We further validated our data using northern blot analysis. For genes with high 5′ UTR coverage, northern blots showed the presence of the full-length transcript in steady-state RNA, but smaller transcript fragments in polysomal RNA (Figure 4C). The presence of these smaller transcript fragments could either indicate a specific enrichment for truncated or non-coding transcripts in polysomal RNA, protection of mRNA by ribosomes or be the result of non-specific degradation of the full-length transcript, although the latter is unlikely considering the high quality of our RNA samples (RNA Integrity Numbers (RINs) are 8.6 and 8.0 for steady state and polysomal total RNA, respectively, and 28S and 18S ribosomal RNA are present in a 2:1 ratio; Figure 4C).

In the 5′ UTR coverage plot, we observed that coverage peaked at the start codon of polysome-associated mRNA (Figure 4A). A similar pattern was previously observed in ribosome-protected fragments from cycloheximide-treated cells [20]. In contrast, apicoplast-encoded genes that are translated in the apicoplast by 70S ribosomes did not show this peak in sequence coverage at the translation start site (Figure 4A). Since 70S ribosomes are insensitive to cycloheximide inhibition [21], excess coverage at the ATG start codon in our polysome-associated mRNA dataset is likely to be the result of cycloheximide-induced ribosome stalling, indicating that even though our mRNA was not nuclease-digested, the binding sites of ribosomes, initiation factors or RNA binding proteins may be enriched in our sequencing data. If binding of ribosomes or proteins indeed influences gene coverage profiles, the high coverage in 5′ UTR regions in this subset of genes may be explained by protection of this region. High 5′ UTR read coverage in this subset of genes may be explained by protection of this region and may thus be the result of the presence of upstream open reading frames (uORFs) that are actively translated. It has been documented that uORFs can limit the translation of the downstream open reading frame (ORF) in a regulated manner [22,23] and that re-initiation capacity of the ribosome is strongly reduced after translating an ORF of 35 or more codons [24]. In line with these data, we observed that a larger proportion of transcripts that showed a translational delay from the trophozoite to the ring stage had a high 5′ UTR coverage (19.0%) compared to transcripts that did not show a translational delay (9.7%) (two-tailed Fisher exact test *P* = 0.005; Figure 4D). Genes with high 5′ UTR coverage and delayed translation also have significantly longer uORFs as compared to genes with low 5′ UTR coverage and no delay in translation (*P* = 0.008; Figure 4E). Although translation of uORFs may activate the nonsense-mediated mRNA decay pathway [25-27], we did not observe a difference in mRNA half-life [28] between transcripts with or without evidence of active uORF translation (data not shown). Collectively, these results point towards translational repression and temporal regulation in *P. falciparum* by uORFs.

### Stop codon readthrough

Similar to the 5′ UTR, we observed higher average gene coverage in the 3′ UTR region in the polysome-associated mRNA as compared to steady-state mRNA (Figure 4F). While a bias towards the 3′ end of transcripts could be introduced by library preparation, the use of both oligo-dT and random hexamer primers during cDNA preparation should have minimized this effect. Furthermore, as this differential pattern was observed in polysomal mRNA but not in steady-state mRNA, it is likely to be biologically relevant. Since higher coverage may be indicative of translational activity, we studied the coding potential of the 3′ UTR regions. In particular, we searched for the presence of in-frame ORFs that could be translated as a result of stop codon readthrough. Numerous stop codon readthrough gene candidates have been identified in other eukaryotes, some of which have been experimentally verified [29-34]. In addition, multiple double-readthrough gene candidates have been detected in *Drosophila* and other metazoa [32], indicating that this might be a common event in eukaryotic genomes. In *P. falciparum*, we identified 133 genes with a substantial ORF (>195 nucleotides) directly downstream of the stop codon. In addition, we found another 85 genes with large downstream ORFs that had a second stop codon within the first 10 codons downstream of the annotated stop codon. The average 3′ UTR coverage for these stop codon readthrough candidates was slightly increased as compared to genes with an ORF smaller than 75 nucleotides (Figure 4G), suggesting that ribosome binding and thus translation of the 3′ UTR may indeed occur for these genes.

To validate our finding, we studied one of the double-readthrough candidates in more detail. PF3D7_1345500, a ubiquitin conjugating enzyme, encodes an annotated gene product of 278 amino acids, including a 28 amino acid signal peptide that is cleaved off after translocation of the protein to the apicoplast [35]. A double-stop codon readthrough event would result in a protein that is 142 amino acids longer (Figure S4A in Additional file 2). Importantly, our polysome sequencing data confirmed that this potential second ORF was part of the full-length transcript and was highly covered by sequence reads (Figure S4B in Additional file 2). Enrichment of the 3′ UTR in polysomal samples was validated by RT-PCR on an independent biological replicate (Figure S4C in Additional file 2). For this gene, three protein bands were observed by western blot analysis using a specific antibody, of which the lowest molecular weight band corresponded to the expected protein size of 33 kDa [35]. Interestingly, the highest band exactly matches the size of a potential double-readthrough product (51 kDa), while the middle band of approximately 48 kDa may represent a ubiquitinylated form of the processed protein, although this remains to be experimentally verified. Taken together, these observations suggest that stop codon readthrough occurs in *P. falciparum* and that the currently annotated proteome is incomplete, although this will need to be verified by mass spectrometry and ribosome-footprinting experiments.

### Alternative splice variants

**Figure 5:**
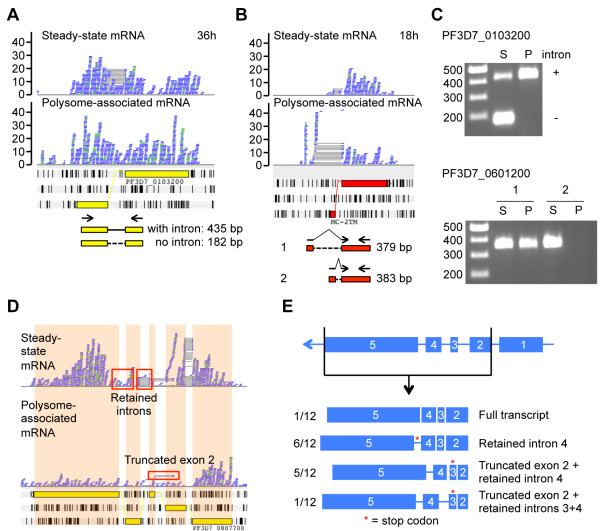
**Alternative splicing in *P. falciparum*. (A)** Intron retention in PF3D7_0103200 observed in polysome-associated mRNA at the schizont stage. **(B)** Differential introns in PF3D7_0601200 in polysomal mRNA as compared to steady-state mRNA, resulting in skipping of exon 1. **(C)** Validation of alternative splice variants in steady-state mRNA (S) and polysomal mRNA (P) fractions by RT-PCR. A schematic representation of the PCR strategy is shown in the lower parts of **(A, B)**. For PF3D7_0103200 (top), the unspliced transcript variant is exclusively detected in polysomal mRNA. For PF3D7_0601200 (bottom), only a non-annotated splice variant is detected in the polysomal fraction. **(D)** Retained introns and truncated exon 2 observed in both steady-state and polysome-associated mRNA for PF3D7_0807700. **(E)** Validation of sequencing results for PF3D7_080770 by cloning of a fragment corresponding to exons 2 to 5 from PF3D7_0807700. Only one out of 12 clones contained the full-length transcript, while the remaining 11 clones encoded truncated versions of the transcript with several combinations of retained intron 3/4 and truncated exon 2.

A genome-wide search for sequence reads that did not match currently annotated splice variants resulted in the discovery of 148 novel introns and alternative splice variants in 125 genes (Table S4 in Additional file 3). Furthermore, we determined that a large fraction of introns (25%) contained five or more mapped reads. A total of 67 highly expressed introns from 60 genes are reported in Table S5 in Additional file 4. Many of these alternative transcripts were exclusively found in steady-state mRNA or polysomal mRNA, indicative of a specialized role for these transcripts in parasite biology. For example, PF3D7_0103200 contains an intron that is spliced out in a large proportion of transcripts detected in steady-state mRNA at 36 h, while removal of this intron is not detected for transcripts associated with polysomes at the same time point (Figure 5A). For PF3D7_0601200, which encodes an MC-2TM protein located in the Maurer’s cleft, we observed the annotated intron in steady-state mRNA at the 18 h time point, while the intron in polysomal mRNA started 289 nucleotides upstream of the regular donor site in the 5′ UTR (Figure 5B). For both genes, these observations were validated by RT-PCR using mRNA from independent biological replicates (Figure 5C).

To further study alternative splicing events, we focused on gene PF3D7_0807700, for which we observed reads mapping to intronic regions as well as reads that mapped to a truncated second exon (Figure 5D). We cloned and sequenced a cDNA fragment from total steady-state RNA corresponding to the region covering exon 2 to exon 5 of gene Pf3D7_0807700. Only one transcript out of 12 clones was identical to the currently known exon model (Figure 5E). The remaining 11 clones consisted of three different alternative transcripts, all of which contained the fourth intron, in some cases in combination with a retained third intron and/or a truncated second exon. These alternative transcripts all contained a premature stop codon, and could thus produce truncated versions of the full-length gene, or be subject to nonsense-mediated mRNA decay.

### Polysome-associated intronic transcripts from *var* genes

The *var* gene family consists of approximately 60 genes each coding for a different variant of the adhesion protein *Plasmodium falciparum* erythrocyte membrane protein 1 (PfEMP1), which is expressed on the surface of the infected erythrocyte. PfEMP1 allows the parasite to adhere to the microvasculature, thus preventing clearance by the spleen, and causing severe disease symptoms associated with cerebral and placental malaria. In addition, the process of antigenic variation, or *var* gene switching, prolongs parasite survival and mediates immune escape. Although each parasite transcribes multiple (possibly all) *var* genes early in its erythrocytic cycle, only one PfEMP1 variant is eventually translated while the remaining variants are silenced [36]. Multiple control mechanisms are likely to be involved in this process of mutually exclusive expression, including upstream and intronic regulatory elements, localization of non-expressed *var* genes in nuclear repressive centers [37-39] and gene silencing by repressive histone marks [40,41]. In addition, two long non-coding RNAs were shown to be transcribed from the intronic regions and to be associated with chromatin [42], which is likely to also contribute to the selective expression of *var* genes. However, these mechanisms are still incompletely understood and a better understanding of *var* gene expression would greatly facilitate novel prevention or intervention strategies for the symptomatic stage of *P. falciparum* infection.

**Figure 6:**
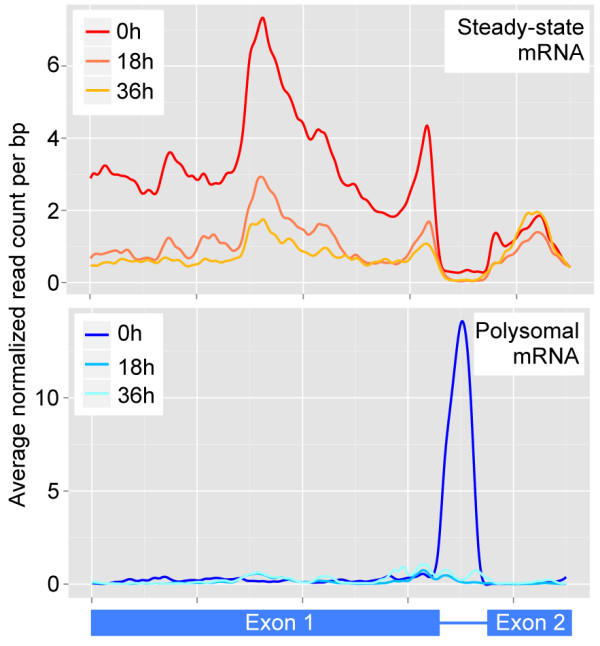
**Average sequence coverage profiles of *var *genes.** High coverage of the exons is observed at the 0 h time point in steady-state mRNA (top), while only intronic reads are detected in polysomal mRNA (bottom).

In line with previous reports [36], we observed that all *var* genes produced transcripts at the 0 h time point within a range of two orders of magnitude (29 to 2,862 read counts per CDS), but that these transcripts were not polysome-associated. Instead, we nearly exclusively observed coverage of *var* introns at the 0 h time point in polysomal mRNA, with an average read count of 161 per intron (Figure 6). Since we used a parasite population with mixed *var* gene expression, we were unable to determine if this pattern was different for the *var* gene that would ultimately be translated by the parasite. Although the expression levels for *var* genes in steady-state mRNA did not directly correlate with the levels of *var* intron read counts in polysomal mRNA, the three *var* variants with the lowest exon coverage (<50 reads per gene) also showed very low intron coverage (≤12 reads per intron). Transcripts encoded by the *var* intronic region have previously been defined as non-coding RNAs. However, an investigation of the coding potential of the *var* introns revealed that both the sense and the antisense intronic sequence contained considerable potential ORFs with average longest ORF lengths of 205 and 155 nucleotides, respectively. Interestingly, the three genes with the lowest exon and intron read counts also contained the shortest potential ORF in the sense intronic sequence. Collectively, these data indicated that control of antigenic variation and translational repression of transcribed var genes also occur at the translational level.

## Discussion

In this study, we aimed to gain a better understanding of mechanisms that control gene expression at the translational level during the asexual cell cycle of *P. falciparum* by comparing next-generation sequencing data from steady-state mRNA and polysome-associated mRNA. We determined that more than 50% of genes expressed during the asexual cycle of the malaria parasite exhibit some form of translational control, ranging from a partial shift in translation levels as compared to transcriptional activity, to a delay in translation of 18 hours or more. The results from this genome-wide analysis confirm and extend previous findings from smaller scale comparisons of the *P. falciparum* transcriptome and proteome [9,10]. In comparison with other eukaryotic genomes, *P. falciparum* encodes relatively few transcription-associated proteins, while it was found to be enriched for proteins involved in chromatin remodeling, translation rates and mRNA stability [6].

Based on these observations, we propose a gene expression model for *P. falciparum* in which the absence of a tight regulatory network for transcription is compensated by post-transcriptional and translational control mechanisms, resulting in a just-in-time translation of proteins. In particular, the parasite seems to have developed a mechanism of delayed translation for proteins that are needed early in the cell cycle, many of which play important roles in erythrocyte remodeling and metabolism. By retaining these transcripts until after re-invasion, the parasite can quickly translate proteins that are critical for its establishment inside the erythrocyte, before it starts a new round of massive transcription and replication. Translational repression by temporary storage of two transcripts in ribonucleoprotein particles has previously been described for the gametocyte stage [12]. The presence of the DDX-6 class RNA helicase DOZI is essential for the formation of these complexes. This protein was recently shown to be present in granular bodies in the cytoplasm of asexual parasites [43], and it is tempting to speculate that DOZI may also be involved in storage of transcripts during the asexual cell cycle.

The wealth of information obtained from RNA-Seq experiments allowed us to subsequently perform an in-depth comparison between these two mRNA subpopulations. We identified major differences in mRNA landscape between steady-state mRNA and polysomal mRNA, which provide important clues for potential regulatory mechanisms. Compared to other eukaryotes, *P. falciparum* genes contain relatively long 5′ UTRs [44]. Recently, the 5′ UTR of the household gene phosphoglucomutase-2 (PGM2; PFD0660w) was shown to play an important role in translation efficiency [45]. In addition, the length and sequence context of the uORF of *P. falciparum var* gene variant *var2csa* was demonstrated to influence the balance between translational repression and translation initiation at the main coding sequence after uORF translation [46]. In line with knowledge from other well-studied eukaryotic organisms [20,22,23,47], uORFs in the 5′ mRNA leaders of *P. falciparum* transcripts are likely to be important regulatory elements that control the level and timing of translation of the main coding sequence. The translation of uORFs by itself does not necessarily influence translation of downstream coding sequences, since the ribosome can continue scanning the mRNA and re-initiate translation at a downstream AUG [20,48,49]. However, the association we observed between uORF translation, translational delay and uORF length suggests that the main inhibitory mechanism of uORFs in *P. falciparum* entails the prevention of ribosomes from reaching the main ORF, possibly by activation of the nonsense-mediated decay pathway [25-27]. Alternatively, many uORFs in the high A/T-biased *P. falciparum* genome contain poly-A tracts that encode lysine repeats, which may significantly slow down the translating ribosome [50]. Furthermore, it is known that uORFs can code for functional peptides that can even exert translational control by themselves [51]. Future studies will have to elucidate the exact nature of these uORFs and their impact on translation.

Another interesting feature of translation that had not been previously described in *P. falciparum* is stop codon readthrough. A recent in-depth study in *Drosophila* classified about 2% of all genes as stop codon readthrough candidates [32], indicating that this may be a relatively common event in eukaryotes. Although further studies will need to validate the occurrence of stop codon readthrough at similar levels in *P. falciparum*, this process could possibly explain the unexpected large size of at least one protein. Phylogenetic analysis of evolutionary constraints on 3′ UTRs and computational methods for the identification of coding regions could shed more light on this mechanism, although currently available tools may have to be adapted for the highly A/T-rich genome of *P. falciparum*. In this respect, it is also interesting to mention that relatively long 3′ UTRs, as frequently observed in *P. falciparum* transcripts, may also harbor binding sites for long non-coding RNAs (lncRNAs) that could influence translation efficiency, similar to what is observed in mammalian neuronal tissues [52].

We detected novel alternative splice variants in the asexual cell cycle of *P. falciparum*, thereby expanding the number of alternative splice variants that are currently annotated or have previously been described in multiple independent RNA-Seq datasets from the same stages [4,18,53]. In addition, we also observed that a large proportion of genes contained sequence reads that mapped to introns. Since the majority of introns were completely devoid of reads, this is unlikely to be caused by DNA contamination of our mRNA samples. Intron coverage can be the result of intron retention in the transcript, or the transcription of overlapping RNAs, either in a sense or anti-sense direction, as is known to occur in *P. falciparum*[53,54]. Alternatively, introns are known to contain many non-protein-coding RNAs (ncRNAs) [55], that can be independent transcripts or be derived from the pre-mRNA. While a number of small nucleolar RNAs (snoRNAs), RNAs of unknown function (RUFs) and other ncRNAs encoded by intronic regions have previously been described for the *P. falciparum* genome [56,57], the widespread detection of intronic coverage is suggestive of a much larger number of regulatory RNAs encoded by introns. In addition, the preferential association of some intronic transcripts with polysomal fractions may point to functions in translational regulation. The apparent role of intron-encoded transcripts in translational repression of the *var* genes demonstrates the potent regulatory function of such RNAs, although the exact mechanism of control remains to be determined. The lack of coverage in the exons suggests that the intronic transcript involved in translational repression is distinct from the two *var* intronic ncRNAs that have previously been described and that either partially overlapped exon 1 or completely overlapped exon 2 [42]. Instead, it may suggest that the intron itself is retained and functional after splicing. Given the purity of our polysome fractions (<2.0% protein contamination), it is unlikely that intronic transcripts were obtained by co-purification of other protein-RNA complexes. Furthermore, even though ribosomes are known to be sticky complexes, the high yield of intronic transcripts in polysomal fractions for virtually all *var* gene variants at the ring stage suggests that this is not the result of mere non-specific adherence, but of specific targeting of intronic *var* transcripts to ribosomes. A better understanding of the role of intronic transcripts or intron-encoded peptides in translational repression of the *var* genes would contribute to strategies for disrupting the mutually exclusive *var* gene expression, thus preventing escape of the parasite from adaptive immune responses.

## Conclusions

Collectively, this study has shown that the regulation of translation in *P. falciparum* is a multi-faceted process of high complexity. Several control mechanisms that have previously been described in higher eukaryotes are also likely to be active in the malaria parasite, including translational repression by upstream ORFs, widespread transcription of non-coding transcripts, and alternative splicing events. This study clearly demonstrates that steady-state mRNA levels are often not predictive for translational activity and that translated transcript variants may differ from the general mRNA population, as recently also shown for human cells [58], indicating that the compartment of actively translated transcripts should not be overlooked. A detailed understanding of the regulatory network that determines gene expression in the different stages of the *P. falciparum* life cycle will not only increase our knowledge of parasite biology, but may ultimately result in the identification of novel antimalarial drug targets.

## Materials and methods

### Parasite culture

The *P. falciparum* strain 3D7 was cultured in human O^+^ erythrocytes at 5% hematocrit as previously described [59]. Cultures were synchronized twice at ring stage with 5% D-sorbitol treatments performed 8 hours apart [60]. Cultures (8% parasitemia in 5% hematocrit in a total volume of 25 ml) were harvested 48 hours after the first sorbitol treatment (ring stage), and then 18 hours (trophozoite stage) and 36 hours thereafter (schizont stage).

### Total RNA extraction

Total RNA was isolated from parasites by adding 5 volumes of pre-warmed Trizol LS Reagent (Life Technologies, Carlsbad, CA, USA) to pelleted infected erythrocytes, followed by a 5 minute incubation at 37°C. RNA isolation was then continued according to the manufacturer’s instructions.

### Polysome-associated RNA isolation

Polysomes were isolated from *P. falciparum* according to a recently published protocol [16]. Briefly, cycloheximide was added to parasite-infected red blood cell cultures to a final concentration of 200 μM, followed by a 10 minute incubation at 37°C. Erythrocytes were then pelleted (4 minutes at 660 x g) and washed twice in phosphate-buffered saline containing 200 μM cycloheximide. After the last wash, pellets were kept on ice and were subsequently lysed by adding 2.2 volumes of lysis buffer (1% (v/v) Igepal CA-360 and 0.5% (w/v) sodium deoxycholate in polysome buffer (400 mM potassium acetate, 25 mM potassium HEPES pH 7.2, 15 mM magnesium acetate, 200 μM cycloheximide, 1 mM dithiothreitol (DTT), and 1 mM 4-(2-aminoethyl)benzenesulfonyl fluoride HCl (AEBSF))). After a 10 minute incubation on ice, lysates were centrifuged for 10 minutes at 20,000 x g at 4°C. The clarified lysates were then loaded on top of a sucrose cushion (1 M sucrose in polysome buffer) to concentrate the ribosomes. For large cultures volumes, 20 ml lysate was loaded on top of 6 ml of sucrose cushion in 26 ml polycarbonate ultracentrifuge tubes and then centrifuged for 3 h at 50,000 rpm at 4°C in a Type 70 Ti rotor (Beckman Coulter, Brea, CA, USA). For small culture volumes, 4 ml lysate was loaded atop 1.25 ml of sucrose cushion in 5 ml polyallomer ultracentrifuge tubes and then centrifuged for 123 minutes at 50,000 rpm at 4°C in an SW 55 Ti rotor (Beckman Coulter). Ribosome pellets were resuspended in polysome buffer, incubated for at least 30 minutes at 4°C to allow complete ribosome resuspension and centrifuged for 10 minutes at 12,000 x g at 4°C. The ribosome suspension was layered on top of a 4.5 ml continuous linear 15 to 60% sucrose (w/v) gradient in polysome buffer and centrifuged for 1.5 h at 50,000 rpm at 4°C in an SW 55 Ti rotor. Fractions of 400 μl were collected using an UA-5 UV detector and model 185 gradient fractionator (ISCO, Lincoln, NE, USA). Polysome fractions were digested with 200 μg Proteinase K (New England Biolabs (NEB), Ipswich, MA, USA) for 1 h at 37°C. RNA was extracted with acid-phenol:chloroform:isoamylalcohol, pH 4.5 (Life Technologies), extracted twice with chloroform and then precipitated using isopropanol.

### Multidimensional protein identification technology

Pooled polysome fractions from a mixed-stage *P. falciparum* culture were analyzed for protein content using MudPIT. Proteins were precipitated with 20% trichloroacetic acid (TCA). The resulting pellet was washed once with 10% TCA and twice with cold acetone. TCA-precipitated protein pellet (approximately 50 μg) was solubilized in Tris–HCl pH 8.5 and 8 M Urea. TCEP (Tris(2-Carboxylethyl)-Phosphine Hydrochloride; Thermo Fisher Scientific, Rockford, IL, USA) and CAM (chloroacetamide; Sigma-Aldrich, St. Louis, MO, USA) were added to a final concentration of 5 mM and 10 mM, respectively. The protein suspension was digested overnight at 37°C using Endoproteinase Lys-C at 1:50 w/w (Roche, Basel, Switzerland). The sample was brought to a final concentration of 2 M urea and 2 mM CaCl_2_ before performing a second overnight digestion at 37°C using Trypsin (Promega, Madison, WI, USA) at 1:100 w/w. Formic acid (5% final) was added to stop the reactions. The sample was loaded on split-triple-phase fused-silica micro-capillary column [61] and placed in-line with LQT Velos Pro mass spectrometer (Thermo Fisher Scientific), coupled with quaternary Agilent 1260 series high-performance liquid chromatography. A fully automated 10-step chromatography run (for a total of 20 hours) was carried out, as described in [62]. Each full mass spectrometry (MS) scan (400 to 1,600 m/z) was followed by 10 data-dependent tandem MS (MS/MS) scans. The number of the micro scans was set to 1 for both MS and MS/MS. The dynamic exclusion settings used were as follows: repeat count 2; repeat duration 30 s; exclusion list size 500 and exclusion duration 90 s, while the minimum signal threshold was set to 500. The MS/MS dataset was searched using SEQUEST [63] against a database of 72,358 sequences, consisting of 5,487 *P. falciparum* non-redundant proteins (downloaded from PlasmoDB on 12 July 2012), 30,536 *H. sapiens* non-redundant proteins (downloaded from NCBI on 27 August 2012), 177 usual contaminants (such as human keratins, IgGs, and proteolytic enzymes), and, to estimate false discovery rates, 36,179 randomized amino acid sequences derived from each non-redundant protein entry. To account for alkylation by CAM, 57 Da were added statically to cysteine residues. To account for the oxidation of methionine residues to methionine sulfoxide (that can occur as an artifact during sample processing), 16 Da were added as a differential modification to methionine residue. Peptide/spectrum matches were sorted, selected using DTASelect/CONTRAST [64]. Proteins had to be detected by one peptide with two independent spectra, leading to false discovery rates at the protein and spectral levels of 2.89% and 0.26%, respectively. To estimate relative protein levels and to account for peptides shared between proteins, Normalized Spectral Abundance Factors (dNSAFs) were calculated for each detected protein, as described in [65]. Lists of all proteins that were detected in our sample and individual peptide/spectral counts are provided in Table S1 in Additional file 1. The mass spectrometry proteomics data have been deposited to the ProteomeXchange Consortium [66] via the PRIDE partner repository [67] with the dataset identifier PXD000553. The MS .RAW files, .ms2 files created by RawDistiller, the .sqt files generated by SEQUEST, and the DTASelect output files for this analysis are also available to download from The Stowers Institute Original Data Repository [68].

### mRNA isolation and cDNA preparation

To remove potential DNA contamination, RNA samples were treated twice with 1 U DNase I (Life Technologies) per 10 μg of RNA for 30 minutes at 37°C, followed by inactivation of the DNase I enzyme. The absence of DNA was confirmed by performing a 40-cycle PCR on *P. falciparum* gene PF05_139450 using 200 to 500 ng total RNA as input. Messenger RNA was then purified from total RNA samples using the GenElute mRNA Miniprep kit (Sigma-Aldrich) according to the manufacturer’s instructions.

The preparation of double-stranded cDNA from steady-state mRNA and polysome-associated mRNA samples was adapted from a previously published method [69]. Up to 500 ng of mRNA was diluted 1:5 in RNA storage solution (Life Technologies) and was fragmented by a 50 minute incubation at 98°C. The fragmented mRNA was added to 3 μg of random hexamers (Integrated DNA Technologies, Coralville, IA, USA), 1 μg of anchored oligo(dT)_20_ (Integrated DNA Technologies) and 1 μl 10 mM dNTP mix (Life Technologies) in a total volume of 10 μl. The mixture was incubated for 10 minutes at 70°C and chilled on ice for 5 minutes. Next, a mix of 2 μl 10X RT buffer, 4 μl 20 mM MgCl_2_, 2 μl 0.1 M DTT, 1 μl 40 U/μl RNaseOUT and 1 μl 200 U/μl SuperScript III Reverse Transcriptase (all from Life Technologies) was added. First-strand cDNA was synthesized by incubating the sample for 10 minutes at 25°C, 50 minutes at 50°C, and finally 5 minutes at 85°C. The first-strand cDNA was then purified using Agencourt AMPure XP beads (Beckman Coulter) and eluted in 47 μl of nuclease-free water. Second-strand cDNA was prepared by adding 2 μl 5X first-strand buffer (Life Technologies), 1 μl 0.1 M DTT (Life Technologies), 15 μl second-strand buffer (Life Technologies), 4 μl 10 mM dNTP mix (Life Technologies), 4 μl 10 U/μl *E. coli* DNA Polymerase (NEB), 1 μl 10 U/μl *E. coli* DNA ligase (NEB), and 1 μl 2 U/μl *E. coli* RNase H (Life Technologies) and incubating the mixture for 2 h at 16°C. Finally, double-stranded cDNA was purified using Agencourt AMPure XP beads.

### Library preparations and sequencing

Libraries from steady-state mRNA samples were prepared using the Encore Multiplexing System (NuGEN, San Carlos, CA, USA) according to the manufacturer’s instructions, with the following modifications for the high AT content of the *P. falciparum* genome: the libraries were amplified for a total of 15 PCR cycles (5 cycles of 15 s at 98°C, 30 s at 55°C, 30 s at 62°C followed by 10 cycles of 15 s at 98°C, 30 s at 63°C, 30 s at 72°C) using KAPA HiFi HotStart Ready Mix (Kapa Biosystems, Woburn, MA, USA). Libraries from polysome-associated mRNA samples were prepared using the NEBNext ChIP-Seq Library Preparation kit (NEB) according to the manufacturer’s instructions, with the exception of the use of the KAPA HiFi Hotstart Ready Mix for the amplification of the libraries. Depending on the amount of input DNA, libraries were amplified for a total of 11 to 15 PCR cycles (3 to 5 cycles of 15 s at 98°C, 30 s at 55°C, 30 s at 62°C followed by 8 to 10 cycles of 15 s at 98°C, 30 s at 63°C, 30 s at 72°C). Libraries of steady-state mRNA samples and of polysomal mRNA samples were multiplexed and were sequenced on two separate lanes with a HiSeq 2000 (Illumina, San Diego, CA, USA), generating 50 bp paired-end sequence reads. By multiplexing all libraries of one sample type into one lane, we attempted to minimize differences in cluster generation and other sequencing artifacts between samples of the same type. The selection of library preparation kits for the construction of sequence libraries was solely based on availability. In our hands, we have not noticed any differences or biases between library preparation kits used in this study. RNA-Seq and polysome-Seq sequence reads are available from the Short Read Archive under accession number SRP021890.

### Sequence mapping

The first five bases and the last base were systematically removed from the sequence reads using FastQ Trimmer, part of the FASTX-Toolkit [70]. Contaminating adaptor reads were removed using Scythe [71]. Reads were then trimmed for bases with a quality score below 30, and reads containing any Ns as well as reads shorter than 18 bases were discarded using Sickle [72]. Subsequently, the trimmed sequence reads were mapped to *P. falciparum* genome v9.0 (downloaded from PlasmoDB [73]) using tophat v2.0.3 [74], allowing a maximum of one mismatch per read segment and no insertions or deletions. We removed all reads that were non-uniquely mapped (using Samtools v0.1.18 [75]), not properly paired (Samtools), PCR duplicates (using Picard Tools v1.78 [76]) or mapped to either ribosomal DNA or to DNA encoding transfer RNA (using Bedtools v2.17.0 [77]). The final number of working reads for each library is listed in Table 1.

### Data normalization

For each gene, the number of reads mapping to its exons was calculated (Bedtools). Exon read counts per gene were normalized for GC content and gene length using the open-source Bioconductor R package EDASeq [78]. In our experience, expression values of short genes with low read counts (<5 reads per gene) are highly inflated using this package. To minimize overestimating expression levels of such genes, genes that did not reach five mapped reads at any time point in both steady-state mRNA and polysomal mRNA were removed from the datasets before applying the normalization algorithm. For genes with annotated alternative splice variants, only the first variant (annotated with .1 in its gene name) was included. Non-protein coding transcripts (annotated as ‘transcript’, not ‘mRNA’ in PlasmoDB-9.0_Pfalciparum3D7.gff) and small nuclear RNAs (snRNAs) were also excluded. Next, to normalize the exon read counts to the mRNA levels per parasite, a scaling factor was calculated for each stage based on the mRNA yield per flask of *P. falciparum*-infected culture (Table 1). For each stage, the total number of working reads was divided by the total number of working reads from the smallest library for that sample type (that is, steady-state mRNA or polysomal mRNA), and was subsequently multiplied by the ratio between the mRNA yield per flask for the stage of the smallest library and the mRNA yield per flask for that particular stage. The exon counts per gene were then divided by this scaling factor. The final normalized abundance values were expressed as counts per kilobase of exon model. Finally, for both steady-state mRNA and polysomal mRNA datasets, genes that were not expressed were excluded from further analysis. Non-expressed genes were defined as having <15% of the median counts per kilobase of exon model (18.9 counts for the steady-state mRNA dataset and 4.0 counts for the polysomal mRNA dataset) at all stages. Because of differences in library sizes, RPKM cutoff values differed for each library, but were at least 0.7. An overview of exon read counts before, during, and after the different normalization steps is provided in Additional file 5.

### Gene clustering and gene ontology analysis

Cluster analysis was performed for genes that were differentially expressed during the cell cycle, defined as a more than two-fold change in exon read counts at any time during the cell cycle, in both steady-state mRNA and polysomal mRNA datasets. For both datasets, genes were subsequently clustered based on scaled expression levels (z-score) using the k-means clustering algorithm with a maximum of 1,000 iterations in R v2.14.2. Several independent clustering runs were performed with increasing numbers of clusters. Determination of the optimal number of clusters was guided by the percentage of variance that was captured by the clusters. We selected the smallest number of clusters that captured more than 90% of the variance (expressed as within group sum of squares) and for which an increase in clusters did not yield a cluster with a novel expression profile (as determined by eye). For both steady-state mRNA and polysomal mRNA, more than 90% of variance could be explained by five or more clusters. Adding a sixth cluster to the polysomal mRNA dataset resulted in a novel cluster (B.6; genes expressed at ring and schizont stage) that was not observed with five or less clusters. The optimal number of clusters was therefore determined to be five clusters for the steady-state mRNA dataset and six clusters for polysomal mRNA dataset. GO analysis was performed for each cluster using the Bioconductor R package goseq [79]. Enriched GO terms were identified using a false discovery rate cutoff of 0.05.

### UTR coverage

Only genes that are located at least 1,000 bp from neighboring genes were included in analyses of 5′ UTR and 3′ UTR coverage. The extent of 5′ UTR coverage was calculated as the ratio between the number of reads that map to the first 500 bp upstream of the start codon and the number of reads that mapped to the coding sequence (expressed per 500 bp to correct for gene size). The numbers of reads mapping to the different gene regions are provided in Additional file 5.

### Coverage plots

Coverage plots were prepared by extracting the normalized read counts for the region of interest for all genes included in the analysis, scaling the read counts for each gene (z-score) and subsequently calculating the average value for each nucleotide position. Coverage profiles were smoothed in R using the function smooth.spline with a smoothing parameter of 0.35, and were subsequently plotted using bioconductor R package ggplot2. For the *var* genes, normalized read counts for exon 1, intron, and exon 2 were extracted separately and were divided into bins of approximately equal length (that is, 650 bins for exon 1, 100 bins for intron and 150 bins for exon 2). The average coverage of each bin was calculated and used for subsequent scaling and averaging across the total length of all *var* genes.

### Semi-quantitative reverse transcription PCR

Reverse transcription was performed for unfragmented steady-state or polysome-associated mRNA using random hexamers and oligo-dT(20) as described above. For directional reverse transcription, three separate cDNA synthesis reactions were performed using 2 pmole of either a forward or a reverse gene-specific primer, or no primer as a control for self-priming (primers are listed in Table S6 in Additional file 2). Subsequently, semi-quantitative PCRs were performed using the KAPA HiFi Hotstart Ready Mix supplemented with 10 ng of cDNA and 10 pmole of both forward and reverse primers. DNA was amplified by incubation for 5 minutes at 95°C, followed by 35 cycles of 30 s at 98°C, 30 s at 55°C, 30 s at 62°C. Samples of 5 μl obtained after cycles 25, 30 and 35 were analyzed by agarose gel electrophoresis. For each primer set, a separate amplification reaction using genomic DNA was performed to control for differences in PCR efficiency.

### Northern blotting analysis

Samples analyzed by northern blot were obtained from independent biological experiments as replicates of sequenced samples. Each RNA sample was loaded in two concentrations, containing 2 μg (left lane) and 8 μg (right lane) of total RNA, respectively. RNA was separated on a 1.2% formaldehyde-agarose gel for 3.5 hours at 40 V. After rinsing the gel twice for 15 minutes in 20X SSC, RNA was transferred for 2.5 hours to Hybond N + membrane (GE Healthcare, Waukesha, WI, USA) using Northern Max Transfer Buffer (Life Technologies), according to the manufacturer’s instructions. After transfer, RNA was cross-linked to the membrane by UV exposure. RNA detection was performed using the DIG Northern Starter Kit (Roche) according to the manufacturer’s instructions with minor modifications for the highly A/T-rich *P. falciparum* genome. Briefly, PCR products were amplified in advance using primers that included the sequence of the SP6 polymerase promoter (primers are listed in Table S6 in Additional file 2). DIG-labeled RNA probes were prepared by incubation of the PCR product with SP6 polymerase in the presence of DIG-labeled nucleotides for 2 hours at 42°C. RNA probes were diluted in ethanol, titrated, stored at -20°C and boiled for 5 minutes just before use. RNA blots were blocked for 30 minutes at 50 to 55°C in pre-warmed 1X DIG Easy Hyb solution and were then incubated O/N at 50 to 55°C in pre-warmed 1X DIG Easy Hyb buffer supplemented with 100 ng/ml of DIG-labeled probe. Blots were washed twice for 5 minutes in 2X SCC, 0.1% SDS at room temperature under constant agitation, followed by two 15 minute washes in pre-warmed 0.1X SSC, 0.1% SDS at 50 to 55°C under constant agitation. After these stringency washes, blots were rinsed in washing buffer, incubated for 30 minutes in blocking solution, incubated for 30 minutes in antibody solution, followed by two 15 minute washes in washing buffer and a 2 minute equilibration in detection buffer. Blots were developed using CDP-Star solution, and were exposed to X-ray film for approximately 25 minutes.

### Analysis of coding potential

The coding potential of a region in the genome (5′ UTR, intron, or 3′ UTR) was determined by scanning for ORFs in all three translation frames. Out of all possible ORFs, only the longest was recorded. For *var* gene introns, this analysis was repeated for the antisense strand.

### Detection of unannotated introns and alternative splice variants

The intron/exon boundaries of all intron-spanning reads (defined by samtools CIGAR string dMdNdM where d is one or more digits) were compared to annotated intron/exon boundaries and were selected for further inspection when one or both intron boundaries were different from current PlasmoDB (version 9.0) annotations. All introns with one unannotated intron/exon boundary supported by at least 5 sequence reads and all novel introns (both intron/exon boundaries unannotated) with at least 10 supporting reads were manually verified in a genome browser and were compared to previously reported alternative splice variants [4,18,53].

### Analysis of intronic reads

Analysis of intronic reads was performed for genes without annotated alternative splice variants. For each intron, we determined the number of intron-spanning reads and the number of sequence reads for which at least 90% of the read length mapped to the intron itself. The ratio of intronic versus intron-spanning reads was then calculated by dividing the number of intronic reads per 100 nucleotides of intron length by the number of intron-spanning reads (one read was added to this number for calculation purposes). To reduce the chance that reads mapping to an intron were derived from an overlapping neighboring gene, only genes that are located at least 750 bp from their nearest annotated neighboring genes were analyzed. Introns with at least 10 reads per 100 nucleotides and at least twice the number of intronic reads versus intron-spanning reads were reported. The numbers of intronic and intron-spanning reads are provided in Additional file 5.

## Abbreviations

bp: Base pair; CAM: Chloroacetamide; CDS: Coding sequence; GO: Gene ontology; MS: Mass spectrometry; MS/MS: Tandem mass spectrometry; MudPIT: Multidimensional protein identification technology; ncRNA: Non-protein-coding RNA; NEB: New England Biolabs; ORF: Open reading frame; TCA: Trichloroacetic acid; uORF: Upstream open reading frame; UTR: Untranslated region.

## Competing interests

The authors declare that they have no competing interests.

## Authors’ contributions

EMB generated sequence data, analyzed the data and wrote the manuscript. DWDC and NP generated sequence data and edited the manuscript. MH performed cloning experiments and edited the manuscript. AS and LF analyzed protein samples by mass-spectrometry (MudPIT). JP maintained parasite cultures and edited the manuscript. KGLR designed the study, supervised the project and wrote the manuscript. All authors read and approved the final manuscript.

## Supplementary Material

Additional file 1 MudPIT analysis of polysome fractions. Click here for file

Additional file 2: Figure S1 Validation of sequencing data. **Figure S2.** Distribution of normalized read counts per gene for the ring, trophozoite and schizont stages of *P. falciparum*. **Figure S3.** Characterization of genes with high 5′ UTR coverage in polysome-associated mRNA. **Figure S4.** Stop codon readthrough candidate in *P. falciparum*. **Table S2.** Enriched gene ontology terms for steady-state mRNA expression clusters. **Table S3.** Enriched gene ontology terms for polysomal mRNA expression clusters. **Table S6.** Sequences of primers used for PCR and northern blot analyses. Click here for file

Additional file 3 Novel introns and alternative splice variants detected in this study. Click here for file

Additional file 4 Highly expressed introns. Click here for file

Additional file 5 **Raw and normalized exon counts for all genes, as well as numbers of reads mapped to the regions 500 bp upstream and 500 bp downstream of each gene.** For genes containing introns, the number of reads mapped to each intron and the number of intron-spanning reads are also provided. Click here for file
