# Supplementary material for: Polysome profiling reveals translational control of gene expression in the human malaria parasite Plasmodium falciparum
Source: Genome Biol. 2013 Nov 22;14(11):R128. doi: 10.1186/gb-2013-14-11-r128 (PMC4053746; doi:10.1186/gb-2013-14-11-r128)
Supplement: Additional file 2: Figure S1 — Validation of sequencing data. Figure S2. Distribution of normalized read counts per gene for the ring, trophozoite and schizont stages of P. falciparum. Figure S3. Characterization of genes with high 5′ UTR coverage in polysome-associated mRNA. Figure S4. Stop codon readthrough candidate in P. falciparum. Table S2. Enriched gene ontology terms for steady-state mRNA expression clusters. Table S3. Enriched gene ontology terms for polysomal mRNA expression clusters. Table S6. Sequences of primers used for PCR and northern blot analyses. [file gb-2013-14-11-r128-S2.pdf]

Supplementary Figures and Tables to:

Polysome profiling reveals translational control of gene expression in the human malaria parasite *Plasmodium falciparum*

Evelien M. Bunnik[1], Duk-Won Doug Chung[1], Michael Hamilton[1], Nadia Ponts[1,2], Anita Saraf[3], Jacques Prudhomme[1], Laurence Florens[3] and Karine G. Le Roch[1]*

[1]Department of Cell Biology and Neuroscience, University of California Riverside, 900 University Ave, Riverside, CA 92521, USA.

[2]Current address: INRA Centre de Bordeaux Aquitaine, Villenave d'Ornon Cedex, France.

[3]Stowers Institute for Medical Research, Kansas City, MO 64110, USA.

* Corresponding author. Email: karine.leroch@ucr.edu

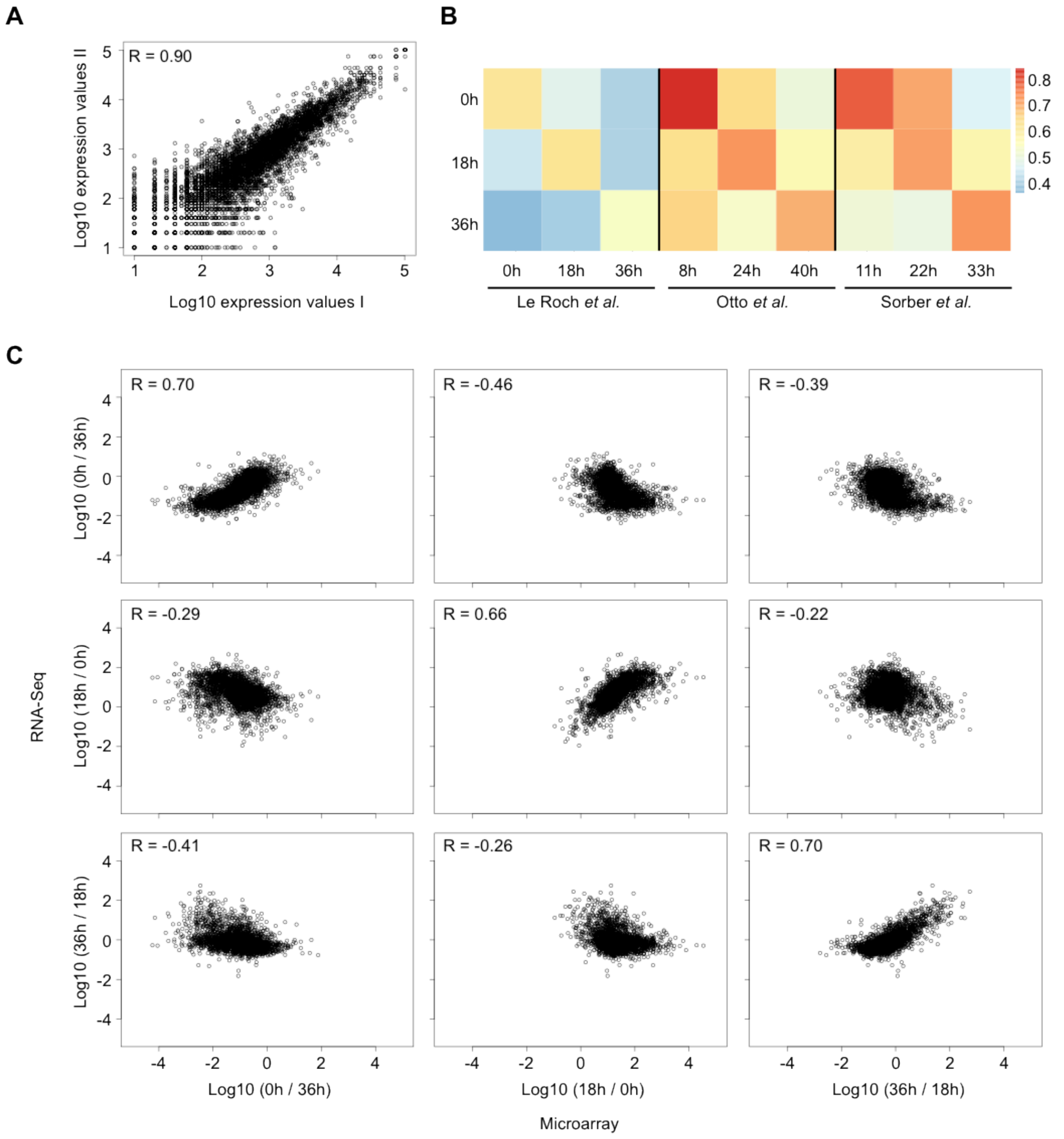

Supplementary Figure 1: Validation of sequencing data. **A**: Correlation between expression values of two biological replicates of polysome-associated mRNA obtained at the 36h time point. (**B**) Pearson correlations between log10-transformed gene expression values for our steady-state mRNA-Seq dataset and previously published microarray (Le Roch et al. 2003) and RNA-Seq datasets (Otto et al. 2010; Sorber et al. 2011). (**C**) Pearson correlations between changes in steady-state mRNA levels obtained previously by microarray (Le Roch et al. 2003) and by RNA-Seq (this study) during the erythrocytic cycle. Changes in expression values between time points were expressed as the log10 fold change. The ring stage expression values obtained by microarray were divided by 10 to correct for the low amount of mRNA present in the parasite at that time point.

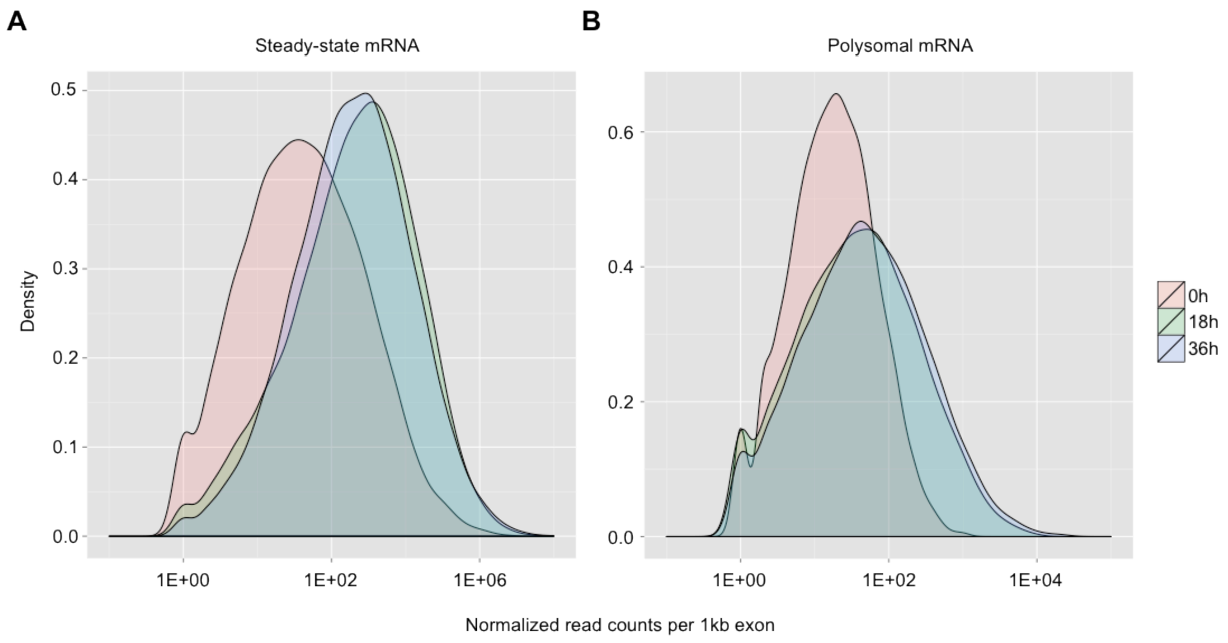

Supplementary Figure 2: Distribution of normalized read counts per 1kb for each gene in the ring, trophozoite, and schizont stages of *P.falciparum*. Gene expression distribution is shown for both steady-state mRNA (A) and polysomal mRNA (B) datasets.

A

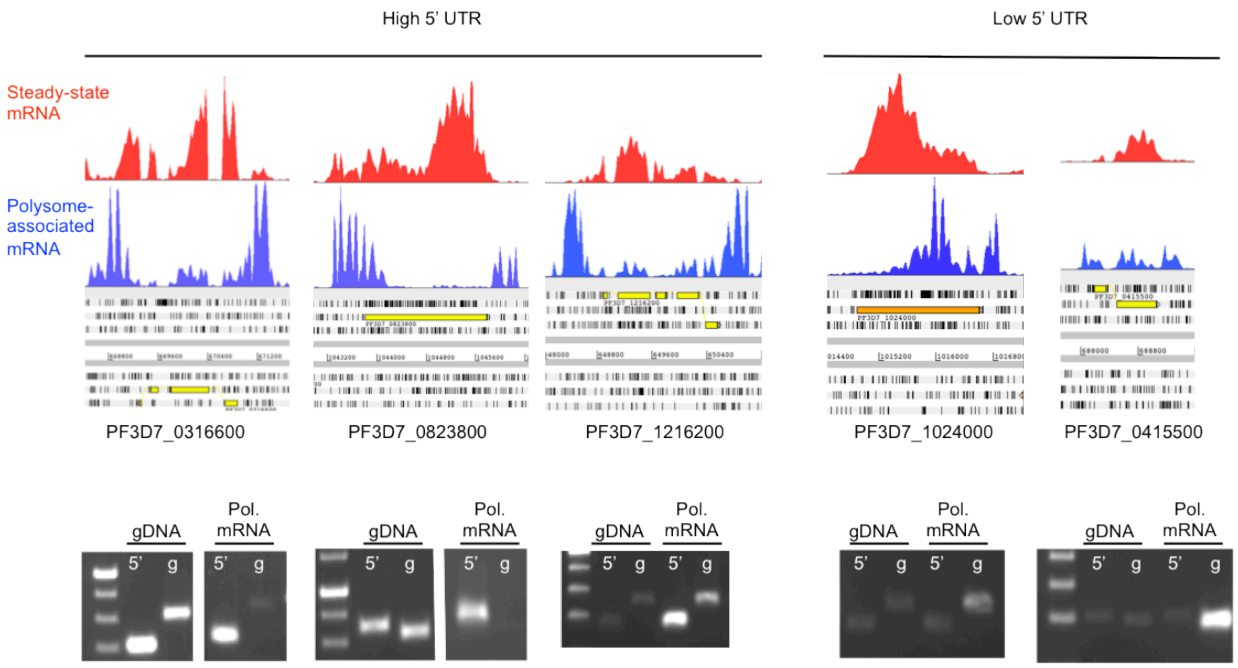

B

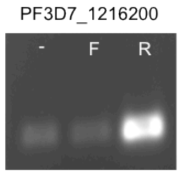

Supplementary Figure 3: Characterization of genes with high 5'UTR coverage in polysome-associated mRNA. **A**: Semi-quantitative PCR amplification of the 5'UTR (5') and gene body (g) of three genes with high 5'UTR coverage and two control genes with low 5'UTR coverage in polysomal mRNA, using cDNA prepared from trophozoite-stage polysome-associated mRNA as input. Samples were obtained from independent biological experiments as replicates of sequenced samples. For each primer set, a separate amplification reaction using genomic DNA (gDNA) was performed to control for differences in PCR efficiency. **B**: PCR amplification of cDNA prepared from polysome-associated mRNA using a forward (F), a reverse (R), or no (-) gene-specific primer for the 5'UTR.

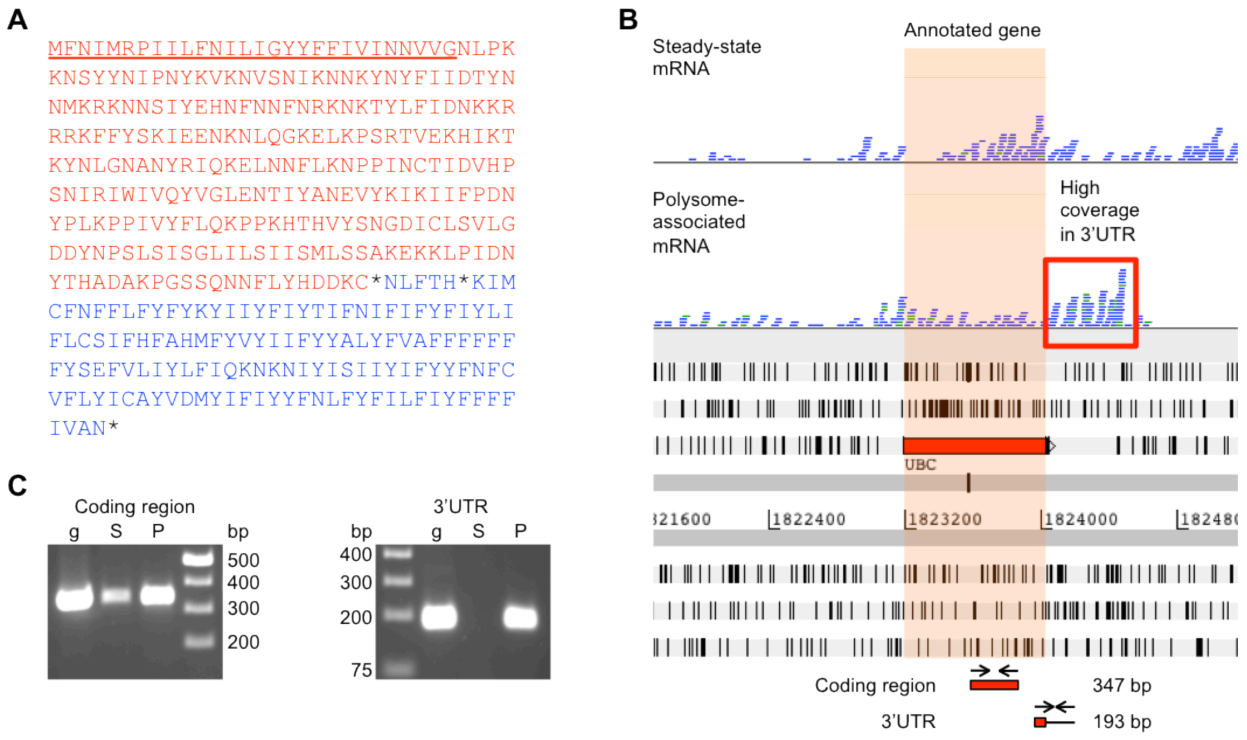

Supplementary Figure 4: Stop codon readthrough candidate in *P. falciparum*. **A**: Amino acid (aa) sequence of the main coding sequence (red) and the ORF directly downstream of the stop codon (blue) of PF3D7_1345500. The apicoplast signaling peptide (28 aa) is underlined. **B**: Genome browser view of steady-state mRNA (top) and polysome-associated mRNA (bottom) sequence coverage for PF3D7_1345500, showing increased coverage of a relatively long 3'UTR region in polysome-associated mRNA. **C**: Enrichment of transcript covering 3'UTR in polysomal (P) sample as compared to a steady-state (S) sample. As a control on PCR efficiency, genomic DNA (g) was included as well. RT-PCR experiments were performed on independent biological replicates. A schematic overview of the PCR strategy is shown in the bottom of panel B.

Supplementary Table 1: MudPIT analysis of polysome fractions.

Supplied as a separate file

Supplementary Table 2: Enriched gene ontology terms for steady-state mRNA expression clusters.

Cluster	Stage	GO term	p-value
A.2	18h	nucleic acid binding	7.06E-07
		translocation of peptides or proteins into host	1.12E-06
		aminoacyl-tRNA ligase activity	1.92E-06
		tRNA aminoacylation for protein translation	2.69E-06
		helicase activity	2.77E-06
		ATP-dependent RNA helicase activity	2.99E-06
		RNA binding	5.69E-05
		ribosome biogenesis	1.31E-04
		RNA metabolic process	1.63E-04
A.3	18h - (36h)	structural constituent of ribosome	4.91E-13
		ribosome	3.42E-11
		DNA replication	1.18E-10
		translation	4.35E-10
		intracellular	6.24E-09
		cytosolic small ribosomal subunit	1.76E-07
		ATP synthesis coupled proton transport	1.09E-04
		chromosome	1.90E-04
		ubiquitin-dependent protein catabolic process	2.05E-04
A.4	(18h) - 36h	intracellular protein transport	1.58E-05
A.5	36h	attachment of GPI anchor to protein	7.41E-08
		rhoptry	8.11E-08
		actin binding	1.70E-07
		entry into host cell	2.29E-07
		motor activity	2.37E-07
		myosin complex	1.14E-05
		integral to membrane	1.13E-04
		cell-cell adhesion	2.17E-04

Supplementary Table 3: Enriched gene ontology terms for polysomal mRNA expression clusters.

Cluster	Stage	GO term	p-value
B.1	0h	heme biosynthetic process	1.02E-06
B.3	18h - (36h)	structural constituent of ribosome	3.64E-14
		ribosome	7.28E-13
		translation	2.83E-12
		intracellular	8.87E-12
		cytosolic small ribosomal subunit	3.74E-11
		nucleic acid binding	3.43E-08
		cytosolic large ribosomal subunit	7.52E-08
		translational elongation	5.27E-06
		translation elongation factor activity	5.79E-05
		small ribosomal subunit	7.92E-05
		RNA binding	2.41E-04
B.4	(18h) - 36h	ubiquitin-dependent protein catabolic process	8.78E-06
		ATP synthesis coupled proton transport	7.15E-05
		proteasome regulatory particle	7.35E-05
		endopeptidase activity	1.23E-04
		vesicle-mediated transport	1.47E-04
B.5	36h	actin binding	2.70E-06
		rhoptry	5.13E-06
		motor activity	8.70E-06
		nucleosome	1.24E-05
		entry into host cell	1.59E-05
		regulation of immune response	2.07E-05
		myosin complex	2.88E-05
		protein kinase activity	6.55E-05
		immunoglobulin production	7.94E-05
		protein serine/threonine kinase activity	1.03E-04
		attachment of GPI anchor to protein	1.18E-04
		protein phosphorylation	1.30E-04
		calmodulin binding	2.32E-04
		nucleosome assembly	3.32E-04
		integral to membrane	4.28E-04
		symbiont-containing vacuole	4.50E-04

Supplementary Table 4: Novel introns and alternative splice variants detected in this study.

Supplied as a separate file

Supplementary Table 5: Highly expressed introns.

Supplied as a separate file

Supplementary Table 6: Sequences of primers used for PCR and northern blot analysis.

Gene	Location	Direction	Sequence
PF3D7_1216200	5'UTR	Forward	5'-TTGTATTTAATCTTGTACCTT-3'
PF3D7_1216200	5'UTR	Reverse	5'-GGAAAAATCATTATGACAAC-3'
PF3D7_1216200	5'UTR	Reverse	5'-ATTTAGGTGACACTATAGAAGGAAAAATCATTATGACAAC-3'[a]
PF3D7_1216200	Gene	Forward	5'-GTGGATAAGAGATGAATTTG-3'
PF3D7_1216200	Gene	Reverse	5'-GCTTTAGCGTGGCTAGC-3'
PF3D7_0823800	5'UTR	Forward	5'-ACCTTGAAATATATATGAAATTG-3'
PF3D7_0823800	5'UTR	Reverse	5'-GGGATTATTTTCCTTAACGA-3'
PF3D7_0823800	5'UTR	Reverse	5'-ATTTAGGTGACACTATAGAAGGGATTATTTTCCTTAACGA-3'[a]
PF3D7_0823800	Gene	Forward	5'-CCGATACAGATAGTTTATGT-3'
PF3D7_0823800	Gene	Reverse	5'-CAGACTTCTTTACATTTAATG-3'
PF3D7_0316600	5'UTR	Forward	5'-GAATACATACTAAGAGCAGC-3'
PF3D7_0316600	5'UTR	Reverse	5'-CTTGTATATAGGTGTATTTTATG-3'
PF3D7_0316600	Gene	Forward	5'-GTTATGACTATATCATTATTCG-3'
PF3D7_0316600	Gene	Reverse	5'-CTATAATATGTTCATATCCTG-3'
PF3D7_1024000	5'UTR	Forward	5'-GATGTGATGCCAACTGTG-3'
PF3D7_1024000	5'UTR	Reverse	5'-CTGAAGCATAAAATATACATAC-3'
PF3D7_1024000	Gene	Forward	5'-GCATATCCTGATATAGGAG-3'
PF3D7_1024000	Gene	Reverse	5'-TCATCATTATGACTATCATTG-3'
PF3D7_0415500	5'UTR	Forward	5'-ATGTTACATTGTTCTTTAGTAG-3'
PF3D7_0415500	5'UTR	Reverse	5'-GCATTATTATGTATATTATAAATTG-3'
PF3D7_0415500	Gene	Forward	5'-TAGATAACTTAGTTGATCATAC-3'
PF3D7_0415500	Gene	Reverse	5'-GCATCATATGTTGAGCGTTC-3'
PF3D7_0103200	Exon 1	Forward	5'-CGTGACCTCTATAATATATCC-3'
PF3D7_0103200	Exon 2	Reverse	5'-TGCTTAAACATAATATGGCAAC-3'
PF3D7_0601200	Intron junction[b]	Forward	5'-CACATACATAATGTAGTAGAAAT-3'
PF3D7_0601200	Intron junction[c]	Forward	5'-TCTATTTAATAACAATGTAGTAGA-3'
PF3D7_0601200	Exon 2	Reverse	5'-GCATACGAAGATGCATTAAATA-3'
PF3D7_1345500	Gene	Forward	5'-TTGCAGGGTAAAGAATTGAAG-3'
PF3D7_1345500	Gene	Reverse	5'-TCATCACCTAATACACTTAAAC-3'
PF3D7_1345500	Gene (end)	Forward	5'-TTCTATATCATGATGATAAATGTT-3'
PF3D7_1345500	3'UTR	Reverse	5'-TACACATAAAACATGTGTGCAA-3'

[a]Primer used in combination with the forward primer for the same region for the generation of probes for northern blot analysis. The SP6 promoter sequence is underlined.

[b]Non-annotated intron junction, genome coordinates Pf3D7_06:47,243 – 47,625.

[c]Annotated intron junction, genome coordinates Pf3D7_06:47,532 – 47,625.

References

Le Roch KG, Zhou Y, Blair PL, Grainger M, Moch JK, Haynes JD, De La Vega P, Holder AA, Batalov S, Carucci DJ et al. 2003. Discovery of gene function by expression profiling of the malaria parasite life cycle. *Science* **301**(5639): 1503-1508.

Lopez-Barragan MJ, Lemieux J, Quinones M, Williamson KC, Molina-Cruz A, Cui K, Barillas-Mury C, Zhao K, Su XZ. 2011. Directional gene expression and antisense transcripts in sexual and asexual stages of Plasmodium falciparum. *BMC Genomics* **12**: 587.

Otto TD, Wilinski D, Assefa S, Keane TM, Sarry LR, Bohme U, Lemieux J, Barrell B, Pain A, Berriman M et al. 2010. New insights into the blood-stage transcriptome of Plasmodium falciparum using RNA-Seq. *Mol Microbiol* **76**(1): 12-24.

Sorber K, Dimon MT, DeRisi JL. 2011. RNA-Seq analysis of splicing in Plasmodium falciparum uncovers new splice junctions, alternative splicing and splicing of antisense transcripts. *Nucleic Acids Res* **39**(9): 3820-3835.
