# Supplementary material for: Polysome profiling reveals translational control of gene expression in the human malaria parasite Plasmodium falciparum
Source: Genome Biol. 2013 Nov 22;14(11):R128. doi: 10.1186/gb-2013-14-11-r128 (PMC4053746; doi:10.1186/gb-2013-14-11-r128)
Supplement: Additional file 3 — Novel introns and alternative splice variants detected in this study. [file gb-2013-14-11-r128-S3.pdf]

Gene	Chromosome	Intron start	Intron stop	Size/Difference*	Location	Description	Dataset$	comments
PF3D7_0108500	Pf3D7_01	349,035	349,157	122	5'UTR	novel intron	S	
PF3D7_0108500	Pf3D7_01	349,411	349,680	269	gene	novel intron	S	
PF3D7_0108900	Pf3D7_01	362,207	362,319	112	gene	novel intron	S+P	
PF3D7_0201800	Pf3D7_02	88,855	89,094	239	5'UTR	novel intron	S+P	
PF3D7_0202900	Pf3D7_02	139,173	139,372	199	5'UTR	novel intron	S+P	overlaps start codon
PF3D7_0214600	Pf3D7_02	593,926	594,195	269	5'UTR	novel intron	S+P	
PF3D7_0214600	Pf3D7_02	594,060	594,195	135	5'UTR	novel intron	S	
PF3D7_0218000	Pf3D7_02	738,494	738,859	365	5'UTR	novel intron	P	
PF3D7_0219400.1	Pf3D7_02	775451	775638	534	Intron 1	alternative donor site	S+P	
PF3D7_0308200	Pf3D7_03	363947	365642	1424	Intron 1	alternative donor site	S	overlaps start codon
PF3D7_0316300.2	Pf3D7_03	659806	659989	1176	Intron 2	alternative donor site	P	
PF3D7_0316300.2	Pf3D7_03	659823	659989	1193	Intron 2	alternative donor site	S+P	
PF3D7_0320000	Pf3D7_03	838,387	838,522	135	3'UTR	novel intron	S+P	
PF3D7_0403800	Pf3D7_04	211,317	211,449	132	3'UTR	novel intron	S+P	
PF3D7_0403800	Pf3D7_04	211,521	211,655	134	3'UTR	novel intron	S+P	
PF3D7_0406200	Pf3D7_04	329,891	330,020	129	3'UTR	novel intron	P	
PF3D7_0409700	Pf3D7_04	456,588	456,833	245	5'UTR	novel intron	P	
PF3D7_0418000	Pf3D7_04	798550	798721	123	Intron 4	alternative donor site	S	
PF3D7_0418000	Pf3D7_04	798598	798721	171	Intron 4	alternative donor site	S+P	
PF3D7_0418300	Pf3D7_04	822,441	824,013	1,572	5'UTR	novel intron	S+P	
PF3D7_0419700	Pf3D7_04	872,952	873,120	168	5'UTR	novel intron	P	
PF3D7_0424600	Pf3D7_04	1,114,505	1,115,006	501	5'UTR	novel intron	P	
PF3D7_0424600	Pf3D7_04	1,114,508	1,115,006	498	5'UTR	novel intron	S+P	
PF3D7_0500800	Pf3D7_05	44,491	44,646	155	gene	novel intron	P	
PF3D7_0500800	Pf3D7_05	45,792	45,890	98	gene	novel intron	P	
PF3D7_0501300	Pf3D7_05	68,511	68,844	333	5'UTR	novel intron	S+P	
PF3D7_0501400	Pf3D7_05	75,995	76,289	294	gene	novel intron	S	
PF3D7_0501400	Pf3D7_05	76,688	77,497	809	gene	novel intron	S	
PF3D7_0502200	Pf3D7_05	105,803	106,038	235	3'UTR	novel intron	S+P	
PF3D7_0503400	Pf3D7_05	140,087	140,436	349	5'UTR	novel intron	S+P	
PF3D7_0519200	Pf3D7_05	791,537	791,722	185	3'UTR	novel intron	P	overlaps stop codon
PF3D7_0519400	Pf3D7_05	797,926	798,083	157	gene/3'UTR	novel intron	P	overlaps stop codon
PF3D7_0519700	Pf3D7_05	817172	817600	-941	Intron 1	alternative acceptor site	S	
PF3D7_0521300	Pf3D7_05	870,258	870,366	108	gene	novel intron	P	anti-sense transcript
PF3D7_0526200	Pf3D7_05	1,083,982	1,084,137	155	5'UTR	novel intron	P	
PF3D7_0601200	Pf3D7_06	47243	47625	-289	Intron 1	alternative donor site	S+P	overlaps start codon
PF3D7_0608310	Pf3D7_06	347,796	348,006	210	5'UTR	novel intron	P	
PF3D7_0608700	Pf3D7_06	358,222	358,390	168	5'UTR	novel intron	S	
PF3D7_0629500	Pf3D7_06	1,216,025	1,216,268	243	5'UTR	novel intron	S+P	
PF3D7_0702400	Pf3D7_07	101,426	101,642	216	5'UTR	novel intron	S	
PF3D7_0704300	Pf3D7_07	197,098	197,632	534	5'UTR	novel intron	P	
PF3D7_0708800	Pf3D7_07	395,163	395,479	316	5'UTR	novel intron	S+P	
PF3D7_0711000	Pf3D7_07	487,007	487,260	253	3'UTR	novel intron	S+P	
PF3D7_0715700	Pf3D7_07	694,331	694,458	127	5'UTR	novel intron	P	
PF3D7_0720900	Pf3D7_07	901627	901783	12	Intron 1	alternative donor site	P	
PF3D7_0722500	Pf3D7_07	958,323	958,815	492	5'UTR	novel intron	S+P	
PF3D7_0726200	Pf3D7_07	1100239	1100413	8	Intron 1	alternative donor site	S+P	
PF3D7_0731000	Pf3D7_07	1,333,898	1,334,084	186	gene	novel intron	S+P	
PF3D7_0813200	Pf3D7_08	652,948	653,154	206	5'UTR	novel intron	P	
PF3D7_0814000	Pf3D7_08	680,477	680,925	448	5'UTR	novel intron	S+P	
PF3D7_0814200	Pf3D7_08	687,809	687,889	80	gene	novel intron	S+P	
PF3D7_0814200	Pf3D7_08	687,962	688,054	92	gene	novel intron	S+P	
PF3D7_0817900	Pf3D7_08	817,117	817,867	750	5'UTR	novel intron	S+P	
PF3D7_0818900	Pf3D7_08	861,222	861,755	533	gene	novel intron	S	
PF3D7_0823200	Pf3D7_08	1,021,516	1,022,681	1,165	3'UTR	novel intron	S	
PF3D7_0823200	Pf3D7_08	1,021,856	1,022,681	825	3'UTR	novel intron	S+P	
PF3D7_0826700	Pf3D7_08	1,161,709	1,162,011	302	5'UTR	novel intron	S+P	
PF3D7_0831800	Pf3D7_08	1375053	1375230	-32	Intron 1	alternative acceptor site	S	
PF3D7_0831800	Pf3D7_08	1,375,313	1,375,559	246	5'UTR	novel intron	S+P	
PF3D7_0903600.2	Pf3D7_09	169610	169704	n.a.	Intron 4	correction exon model	S+P	novel exon at 169,602-169,609
PF3D7_0905400	Pf3D7_09	274,809	274,926	117	5'UTR	novel intron	S+P	
PF3D7_0907800	Pf3D7_09	366,778	366,880	102	5'UTR	novel intron	S+P	
PF3D7_0908300.1	Pf3D7_09	385171	385351	-4	Intron 2	alternative acceptor site	P	
PF3D7_0918100	Pf3D7_09	747045	747354	-137	Intron 1	alternative donor site	S	overlaps start codon
PF3D7_0918500	Pf3D7_09	763,444	763,706	262	3'UTR	novel intron	S	
PF3D7_0927900	Pf3D7_09	1,132,151	1,133,081	930	5'UTR	novel intron	S+P	
PF3D7_0935900	Pf3D7_09	1,422,081	1,422,155	74	gene	novel intron	S	
PF3D7_0935900	Pf3D7_09	1422436	1422626	8	Intron 1	alternative donor site	S	
PF3D7_1001200	Pf3D7_10	70,854	70,970	116	5'UTR	novel intron	S+P	
PF3D7_1008700	Pf3D7_10	360,800	361,183	383	gene	novel intron	S	
PF3D7_1010300	Pf3D7_10	414,280	414,658	378	5'UTR	novel intron	S+P	
PF3D7_1010300	Pf3D7_10	414969	415102	-35	Intron 1	alternative donor site	S+P	
PF3D7_1012400	Pf3D7_10	475912	476546	n.a.	Intron 3	exon 2 skipping	S	
PF3D7_1015600	Pf3D7_10	629,046	629,874	828	5'UTR	novel intron	S	
PF3D7_1015800	Pf3D7_10	632,902	633,486	584	5'UTR	novel intron	S+P	
PF3D7_1016300	Pf3D7_10	651,629	652,078	449	gene	novel intron	S	
PF3D7_1016300	Pf3D7_10	651,929	652,078	149	gene	novel intron	S	

PF3D7_1019700	Pf3D7_10	802,605	802,685	80	gene	novel intron	P	
PF3D7_1020900	Pf3D7_10	846,202	846,553	351	5'UTR	novel intron	S+P	
PF3D7_1023900	Pf3D7_10	1,008,246	1,009,309	1,063	5'UTR	novel intron	S+P	
PF3D7_1030800	Pf3D7_10	1,249,925	1,250,041	116	5'UTR	novel intron	P	
PF3D7_1031400	Pf3D7_10	1,264,879	1,265,064	185	5'UTR	novel intron	S+P	
PF3D7_1034800	Pf3D7_10	1,380,081	1,380,219	138	5'UTR	novel intron	P	overlaps start codon
PF3D7_1104800	Pf3D7_11	206,889	207,143	254	5'UTR	novel intron	S+P	
PF3D7_1105100	Pf3D7_11	226,521	226,574	53	3'UTR	novel intron	S+P	
PF3D7_1105800	Pf3D7_11	250,636	250,835	199	5'UTR	novel intron	S+P	
PF3D7_1107100	Pf3D7_11	295200	295479	n.a.	Intron 7	exon 4 skipping	S+P	
PF3D7_1110000	Pf3D7_11	398689	399011	4	Intron 1	alternative acceptor site	P	
PF3D7_1112600	Pf3D7_11	481,066	481,227	161	5'UTR	novel intron	P	
PF3D7_1114700	Pf3D7_11	554,644	554,836	192	5'UTR	novel intron	S+P	
PF3D7_1114700	Pf3D7_11	554,929	555,299	370	5'UTR	novel intron	S+P	
PF3D7_1117700	Pf3D7_11	671,530	672,067	537	5'UTR	novel intron	S+P	
PF3D7_1123400	Pf3D7_11	925,592	925,727	135	5'UTR	novel intron	S+P	
PF3D7_1130200	Pf3D7_11	1,164,608	1,164,985	377	5'UTR	novel intron	S+P	
PF3D7_1131200	Pf3D7_11	1199599	1199672	140	Intron 1	alternative donor site	P	
PF3D7_1134000	Pf3D7_11	1,319,631	1,320,093	462	5'UTR	novel intron	S+P	
PF3D7_1136500	Pf3D7_11	1,435,192	1,436,787	1,595	5'UTR	novel intron	S+P	
PF3D7_1139900	Pf3D7_11	1,593,461	1,593,571	110	5'UTR	novel intron	P	
PF3D7_1148900	Pf3D7_11	1,945,408	1,945,651	243	5'UTR	novel intron	S	
PF3D7_1222700	Pf3D7_12	919,342	920,026	684	3'UTR	novel intron	S	
PF3D7_1222700	Pf3D7_12	919,377	920,026	649	3'UTR	novel intron	S	
PF3D7_1224100	Pf3D7_12	977,903	978,472	569	5'UTR	novel intron	S+P	
PF3D7_1228500	Pf3D7_12	1158612	1158708	29	Intron 1	alternative donor site	P	
PF3D7_1236700	Pf3D7_12	1,529,185	1,529,327	142	3'UTR	novel intron	S+P	anti-sense transcript
PF3D7_1237400	Pf3D7_12	1559416	1559723	-131	Intron 2	alternative donor site	S+P	
PF3D7_1237400	Pf3D7_12	1559512	1559723	-35	Intron 2	alternative donor site	S	
PF3D7_1249300	Pf3D7_12	2,015,823	2,015,957	134	3'UTR	novel intron	S+P	
PF3D7_1252300	Pf3D7_12	2,127,845	2,128,135	290	3'UTR	novel intron	S+P	
PF3D7_1253000	Pf3D7_12	2167710	2168412	-534	Intron 1	alternative donor site	S	overlaps start codon
PF3D7_1302100	Pf3D7_13	118,975	119,078	103	3'UTR	novel intron	S	novel gene?
PF3D7_1310700	Pf3D7_13	468,696	468,858	162	5'UTR	novel intron	S+P	
PF3D7_1316200	Pf3D7_13	679,917	680,022	105	5'UTR	novel intron	S	
PF3D7_1321700	Pf3D7_13	905,297	905,924	627	3'UTR	novel intron	S	
PF3D7_1321700	Pf3D7_13	905,633	905,924	291	3'UTR	novel intron	P	
PF3D7_1326300	Pf3D7_13	1,092,127	1,092,295	168	3'UTR	novel intron	S+P	
PF3D7_1330300	Pf3D7_13	1276349	1276739	-31	Intron 5	alternative donor site	S	
PF3D7_1337400	Pf3D7_13	1508142	1508597	n.a.	Intron 3	exon 2 skipping	S	
PF3D7_1340700	Pf3D7_13	1,622,723	1,622,954	231	5'UTR	novel intron	P	
PF3D7_1340900	Pf3D7_13	1,626,150	1,627,253	1,103	5'UTR	novel intron	S+P	
PF3D7_1342000	Pf3D7_13	1,654,138	1,654,485	347	5'UTR	novel intron	S+P	
PF3D7_1346100	Pf3D7_13	1,843,121	1,843,276	155	5'UTR	novel intron	S+P	
PF3D7_1347500	Pf3D7_13	1,899,363	1,899,543	180	5'UTR	novel intron	S+P	
PF3D7_1353600	Pf3D7_13	2,147,268	2,147,445	177	5'UTR	novel intron	P	
PF3D7_1357100	Pf3D7_13	2,266,743	2,269,273	2,530	5'UTR	novel intron	S	
PF3D7_1358700	Pf3D7_13	2,327,014	2,327,609	595	5'UTR	novel intron	S+P	
PF3D7_1360300	Pf3D7_13	2,409,156	2,409,306	150	3'UTR	novel intron	S	
PF3D7_1360300	Pf3D7_13	2,409,156	2,409,310	154	3'UTR	novel intron	S	
PF3D7_1360900	Pf3D7_13	2440705	2440915	-22	Intron 8	alternative donor site	S+P	
PF3D7_1366900	Pf3D7_13	2675853	2676092	-91	Intron 2	alternative donor site	S	
PF3D7_1372200	Pf3D7_13	2,840,780	2,840,840	60	gene	novel intron	S	
PF3D7_1372200	Pf3D7_13	2,841,050	2,841,391	341	gene	novel intron	S	
PF3D7_1372200	Pf3D7_13	2,841,266	2,841,373	107	gene	novel intron	S	
PF3D7_1372200	Pf3D7_13	2841486	2842023	389	Intron 1	alternative donor site	S	overlaps start codon
PF3D7_1372200	Pf3D7_13	2,841,717	2,842,023	306	5'UTR	novel intron	S+P	
PF3D7_1401100	Pf3D7_14	43,108	43,242	134	5'UTR	novel intron	S	
PF3D7_1401600	Pf3D7_14	63,003	63,794	791	5'UTR	novel intron	S+P	
PF3D7_1404900	Pf3D7_14	169,713	169,930	217	5'UTR	novel intron	P	
PF3D7_1406700	Pf3D7_14	244,751	244,907	156	5'UTR	novel intron	P	
PF3D7_1413700	Pf3D7_14	541491	541743	71	Intron 8	alternative donor site	S+P	
PF3D7_1424400	Pf3D7_14	979,168	979,974	806	3'UTR	novel intron	S+P	intron overlaps PF3D7_1424500 and PF3D7_1424600
PF3D7_1433700	Pf3D7_14	1,349,005	1,349,112	107	5'UTR	novel intron	S+P	
PF3D7_1444800	Pf3D7_14	1845148	1845889	275	Intron 1	alternative donor site	S	overlaps start codon
PF3D7_1447000	Pf3D7_14	1930366	1930508	2	Intron 2	alternative acceptor site	S+P	
PF3D7_1449200	Pf3D7_14	2,014,478	2,014,657	179	5'UTR	novel intron	S+P	
PF3D7_1463900	Pf3D7_14	2,589,793	2,589,907	114	5'UTR	novel intron	S+P	
PF3D7_1468200	Pf3D7_14	2,801,697	2,802,457	760	5'UTR	novel intron	S+P	
PF3D7_1469900	Pf3D7_14	2,866,334	2,866,785	451	gene	novel intron	S+P	anti-sense transcript
PF3D7_1476000	Pf3D7_14	3,128,133	3,128,319	186	5'UTR	novel intron	S	

*For alternative donor and acceptor sites, the difference in genomic location is indicated. For novel introns, the length of the intron is given.

$Dataset is indicated as S for steady-state mRNA or P for polysome-associated mRNA
