# Supplementary material for: Polysome profiling reveals translational control of gene expression in the human malaria parasite Plasmodium falciparum
Source: Genome Biol. 2013 Nov 22;14(11):R128. doi: 10.1186/gb-2013-14-11-r128 (PMC4053746; doi:10.1186/gb-2013-14-11-r128)
Supplement: Additional file 4 — Highly expressed introns. [file gb-2013-14-11-r128-S4.pdf]

Gene	Chromosome	Intron start	Intron end	Intron	Dataset*	Stage	Steady-state mRNA		Polysome mRNA	
							intronic reads/100b	overlapping reads	intronic reads/100b	overlapping reads
PF3D7_0103200	PF3D7_01	141,087	141,339	1	P	36h	17	25	24	0
PF3D7_0111000	PF3D7_01	422,497	422,731	3	P	18h / 36h	7	0	111	0
PF3D7_0209100	PF3D7_02	382,878	383,070	1	P	0h	1	0	12	0
PF3D7_0214900	PF3D7_02	614,971	615,180	14	P	0h	0	0	12	0
PF3D7_0314500	PF3D7_03	584,256	584,448	1	S	0h	15	5	0	0
PF3D7_0321200	PF3D7_03	887,660	887,778	5	P	36h	7	1	13	0
PF3D7_0321200	PF3D7_03	887,442	887,591	6	S	36h	11	0	7	0
PF3D7_0404300	PF3D7_04	236,285	236,400	4	P	0h	20	112	10	0
PF3D7_0416000	PF3D7_04	702,569	702,695	2	S	0h	11	4	2	0
PF3D7_0424500	PF3D7_04	1,112,075	1,112,218	2	S	0h	17	6	3	0
PF3D7_0506800	PF3D7_05	287,640	287,940	2	P	18h	1	4	22	4
PF3D7_0525700	PF3D7_05	1,068,768	1,069,141	1	P	36h	5	0	10	0
PF3D7_0528900	PF3D7_05	1,184,750	1,184,915	5	S	0h	10	1	1	0
PF3D7_0705800	PF3D7_07	288,477	288,670	3	P	18h	0	0	13	0
PF3D7_0705800	PF3D7_07	287,804	287,912	6	S	0h	11	0	0	0
PF3D7_0706300	PF3D7_07	305,878	305,990	1	S	0h	58	7	1	0
PF3D7_0726200	PF3D7_07	1,100,231	1,100,413	1	S	0h	50	0	7	0
PF3D7_0806600	PF3D7_08	352,734	353,310	1	S	0h	12	0	0	0
PF3D7_0807300	PF3D7_08	381,628	381,862	1	S	0h	15	0	3	0
PF3D7_0813400	PF3D7_08	659,128	660,157	2	P	18h / 36h	4	103	60	19
PF3D7_0828900	PF3D7_08	1,242,298	1,242,438	3	S	36h	11	3	1	0
PF3D7_0902300	PF3D7_09	104,347	104,467	1	S	0h	12	0	2	0
PF3D7_0911000	PF3D7_09	501,890	502,033	9	S	36h	17	3	1	0
PF3D7_0911000	PF3D7_09	502,074	502,206	10	S	RS	26	2	2	50
PF3D7_0916000	PF3D7_09	667,315	667,424	3	S	0h	15	0	1	0
PF3D7_0922100	PF3D7_09	899,660	899,831	1	P	18h	2	50	19	3
PF3D7_0934600	PF3D7_09	1,354,665	1,354,847	1	S	0h	11	4	1	0
PF3D7_0935500	PF3D7_09	1,401,880	1,402,000	2	S + P	18h / 36h	20	0	37	0
PF3D7_1005800	PF3D7_10	252,129	252,359	1	S	18h	16	0	3	0
PF3D7_1013400	PF3D7_10	520,420	520,623	4	P	18h / 36h	4	2	51	2
PF3D7_1014000	PF3D7_10	552,111	552,220	1	S	18h	13	1	0	0
PF3D7_1017400	PF3D7_10	699,265	699,368	2	S	0h	11	1	0	0
PF3D7_1017400	PF3D7_10	698,812	698,913	4	S	0h	16	3	0	0
PF3D7_1020200	PF3D7_10	817,788	818,136	2	P	18h	1	0	13	0
PF3D7_1027900	PF3D7_10	1,158,399	1,158,513	7	S	0h	17	0	5	0
PF3D7_1037900	PF3D7_10	1,503,517	1,503,703	2	S	0h	11	2	0	0
PF3D7_1103000	PF3D7_11	138,835	138,947	1	S	0h	14	2	1	0
PF3D7_1108500	PF3D7_11	367,684	367,962	2	P	0h	0	0	24	0
PF3D7_1111600	PF3D7_11	450,943	451,224	1	P	0h	0	0	12	0
PF3D7_1125000	PF3D7_11	984,412	984,581	4	S	0h	21	0	1	0
PF3D7_1136900	PF3D7_11	1,458,892	1,459,034	1	S	0h	19	0	0	0
PF3D7_1145300	PF3D7_11	1,798,855	1,799,053	3	P	0h	2	1	12	0
PF3D7_1145300	PF3D7_11	1,799,123	1,799,293	2	P	36h	2	0	14	0
PF3D7_1149500	PF3D7_11	1,993,688	1,993,826	1	P	0h	0	0	12	0
PF3D7_1226400	PF3D7_12	1,067,708	1,067,849	3	S	0h	13	0	0	0
PF3D7_1226400	PF3D7_12	1,067,509	1,067,661	4	S	0h	24	0	0	0
PF3D7_1226800	PF3D7_12	1,085,468	1,085,857	1	S	36h	19	0	8	0
PF3D7_1231700	PF3D7_12	1,306,594	1,306,834	1	S	0h	15	1	0	0
PF3D7_1240900	PF3D7_12	1,741,003	1,742,004	1	P	0h	5	22	18	0
PF3D7_1242400	PF3D7_12	1,796,494	1,796,867	2	P	18h	7	1	13	0
PF3D7_1315400	PF3D7_13	649,263	650,039	3	P	0h / 18h / 36h	74	183	178	10
PF3D7_1315600	PF3D7_13	655,530	655,608	7	S	36h	22	0	0	1
PF3D7_1325200	PF3D7_13	1,048,340	1,048,635	2	P	0h	1	0	17	0
PF3D7_1326200	PF3D7_13	1,090,403	1,090,520	2	S	18h	11	0	3	0
PF3D7_1328600	PF3D7_13	1,211,622	1,211,724	1	P	0h	0	0	12	0
PF3D7_1329400	PF3D7_13	1,245,688	1,245,863	5	S	0h	11	0	0	0
PF3D7_1329400	PF3D7_13	1,246,141	1,246,277	7	S	0h	14	4	4	0
PF3D7_1334500	PF3D7_13	1,407,571	1,407,909	1	P	18h	1	0	18	0
PF3D7_1356900	PF3D7_13	2,264,399	2,264,616	1	S	0h	14	2	1	0
PF3D7_1360700	PF3D7_13	2,427,884	2,429,115	4	P	18h	1	4	30	0
PF3D7_1409400	PF3D7_14	365,913	366,181	3	P	0h	0	12	12	0
PF3D7_1410800	PF3D7_14	435,109	435,246	6	S	18h / 36h	25	0	1	0
PF3D7_1428400	PF3D7_14	1,108,986	1,109,148	6	S	0h	11	0	2	0
PF3D7_1446700	PF3D7_14	1,919,613	1,919,717	7	S	18h / 36h	23	2	4	0
PF3D7_1449100	PF3D7_14	2,012,321	2,012,422	6	S	36h	19	1	0	0
PF3D7_1463900	PF3D7_14	2,584,953	2,585,281	13	P	0h	0	0	13	0
PF3D7_1464600	PF3D7_14	2,623,217	2,623,396	1	S + P	18h / 36h	166	85	262	16

*Dataset is indicated as S for steady-state mRNA or P for polysome-associated mRNA
